# Enhanced In Vitro Antitumor Activity of GnRH-III-Daunorubicin Bioconjugates Influenced by Sequence Modification

**DOI:** 10.3390/pharmaceutics10040223

**Published:** 2018-11-09

**Authors:** Sabine Schuster, Beáta Biri-Kovács, Bálint Szeder, László Buday, János Gardi, Zsuzsanna Szabó, Gábor Halmos, Gábor Mező

**Affiliations:** 1 Faculty of Science, Institute of Chemistry, Eötvös Loránd University, 1117 Budapest, Hungary; sabine.schuster83@gmail.com (S.S.); biri.beata@gmail.com (B.B.-K.); 2 MTA-ELTE Research Group of Peptide Chemistry, Hungarian Academy of Sciences, Eötvös Loránd University, 1117 Budapest, Hungary; 3 Research Centre for Natural Sciences, Institute of Enzymology, Hungarian Academy of Sciences, 1117 Budapest, Hungary; szederbalint@gmail.com (B.S.); buday.laszlo@ttk.mta.hu (L.B.); 4 First Department of Internal Medicine, Faculty of Medicine, University of Szeged, 6720 Szeged, Hungary; gardi.janos@med.u-szeged.hu; 5 Department of Biopharmacy, Faculty of Pharmacy, University of Debrecen, 4032 Debrecen, Hungary; szabo.zsuzsanna@pharm.unideb.hu (Z.S.); halmos.gabor@pharm.unideb.hu (G.H.)

**Keywords:** targeted cancer therapy, drug delivery system, daunorubicin, gonadotropin releasing hormone III, peptide drug conjugates, oxime linkage, antitumor activity, cellular uptake

## Abstract

Receptors for gonadotropin releasing hormone (GnRH) are highly expressed in various human cancers including breast, ovarian, endometrial, prostate and colorectal cancer. Ligands like human GnRH-I or the sea lamprey analogue GnRH-III represent a promising approach for the development of efficient drug delivery systems for targeted tumor therapy. Here, we report on the synthesis and cytostatic effect of 14 oxime bond-linked daunorubicin GnRH-III conjugates containing a variety of unnatural amino acids within the peptide sequence. All compounds demonstrated a reduced cell viability in vitro on estrogen receptor α (ERα) positive and ERα negative cancer cells. The best candidate revealed an increased cancer cell growth inhibitory effect compared to our lead-compound GnRH-III-[^4^Lys(Bu),^8^Lys(Dau=Aoa)]. Flow cytometry and fluorescence microscopy studies showed that the cellular uptake of the novel conjugate is substantially improved leading to an accelerated delivery of the drug to its site of action. However, the release of the active drug-metabolite by lysosomal enzymes was not negatively affected by amino acid substitution, while the compound provided a high stability in human blood plasma. Receptor binding studies were carried out to ensure a high binding affinity of the new compound for the GnRH-receptor. It was demonstrated that GnRH-III-[^2^ΔHis,^3^d-Tic,^4^Lys(Bu),^8^Lys(Dau=Aoa)] is a highly potent and promising anticancer drug delivery system for targeted tumor therapy.

## 1. Introduction

Targeted cancer therapy is a promising tool to overcome the drawbacks of classical chemotherapy like the lack of selectivity, toxicity to healthy tissue and the development of multidrug resistance forced by high dose treatments. In general, ligands with high binding affinities to tumor-specific receptors or receptors which are overexpressed on the surface of cancer cells can be used as carriers for anticancer drugs enabling the selective delivery of an effective cytotoxic agent or radionuclides to tumor cells. Peptide ligands provide valuable properties such as excellent tissue permeability, low immunogenicity and structural simplicity. In comparison to protein-based biopharmaceuticals like monoclonal antibodies, an additional benefit is that peptides can be produced cost-efficiently in large scale by chemical synthesis [1,2]. Especially, the good progress in peptide technology, solid phase synthesis and chemical ligation techniques facilitates the synthesis and modification of peptides [3,4]. The main problem of the application of peptide therapeutics is their relatively short plasma half-life and the corresponding in vivo stability. The proteolytic digestion of the peptides can be prevented or reduced by incorporating unnatural amino acids (e.g., d-amino acids) or peptide cyclization [5,6,7]. Replacing one or more amino acids with their d-version may not only enhance the proteolytic stability but also lead to an improved or reduced activity and/or receptor affinity of the compounds. Prominent examples hereby are gonadotropin-releasing hormone (GnRH) agonists and antagonists like triptorelin and cetrorelix, which have been developed by Schally and coworkers [8,9]. Both peptide-based pharmaceutics were approved by the Food and Drug Administration (FDA) at the beginning of this century [10,11]. Cetrorelix acetate was the first GnRH antagonist on the market and is used to prevent premature luteinizing hormone (LH) surges in women undergoing controlled ovarian stimulation, whereas triptorelin pamoate received approval for the palliative treatment of advanced prostate cancer [10,11,12]. These potent GnRH analogs bind like the natural peptide hormone GnRH-I (<EHWSYGLRPG-NH_2_; <E is pyroglutamic acid) to pituitary GnRH receptor (GnRH-R) on gonadotrophs [13,14]. The native ligand of this seven-transmembrane G-protein coupled receptor regulates and stimulates the synthesis and release of gonadotropins like LH and follicle stimulating hormone (FSH), whereby the application of GnRH analogs causes inhibition of gonadotropin release, which can occur by two different mechanisms of action [12]. Thus, a constant exposure to GnRH agonists triggers a desensitization of the gonadotropic cells as well as the downregulation of the receptor level on the cell surface followed by a reduced LH and FSH release [15]. In contrast, the administration of GnRH antagonist leads to a direct but reversible competitive receptor blockage causing an immediate inhibition of the gonadotropin release [15,16]. In addition, it has been discovered that GnRH-Rs are not only expressed in pituitary but also on the surface of various human tumor cells including breast cancer, prostate cancer, and endometrial cancer [17]. Based on these findings, efficient cytotoxic GnRH-I derivatives were developed in Schally’s laboratory [18,19,20,21,22]. The most prominent conjugate AEZS-108 (Zoptrex^TM^, previously AN-152) consists of a GnRH-I-[^6^d-Lys] targeting moiety and the antitumor agent doxorubicin (Dox), which was conjugated to the side chain of the ^6^d-Lys through an ester bond by insertion of a glutaryl spacer. It has been demonstrated that AEZS-108 internalizes selectively in GnRH-R expressing cells followed by an intracellular release of the drug by tumor-specific carboxylesterases [20]. Thus, the antitumor effect of AEZS-108 was intensively studied in vitro and in vivo revealing a significant tumor growth inhibition and regression of several tumor types in vivo [17,23,24,25]. Due to the positive results, preclinical studies and clinical trials were performed up to phase III. Unfortunately, AEZS-108 could not achieve its primary endpoint in clinical phase III studies on endometrial cancer, which was caused by the lack of a significant difference in the median period of overall survival of patients treated with Zoptrex™ as compared to patients treated with doxorubicin [26]. The main reason for this might be the poor enzymatic stability of the conjugate in circulation. It has been shown that the ester bond can be hydrolyzed rapidly by carboxylesterases in presence of mouse (*t*_1/2_ = 19 min) and human serum (*t*_1/2_ = 126 min) [27]. Taking this into account, drug-linkers with higher enzymatic stability under physiological conditions might help to overcome this weakness and ensure antitumor activity without toxic side effects. A variety of linkage systems like ester, hydrazone, oxime or amide bond have been developed to link anthracyclines like Dox or daunorubicin (Dau) to GnRH-based targeting moieties [28,29,30]. Hereby, the oxime linked drug conjugates revealed the highest chemical and enzymatical stability [30]. 

Similarly to GnRH-I derivatives, GnRH-III (<EHWSHDWKPG–NH_2_) based peptide carriers have been also used in our laboratories to deliver anticancer agents like Dau selectively to GnRH-R expressing cancer cells [28,29,30,31]. This native GnRH-I analog, isolated from sea lamprey, binds to GnRH-R on cancer cells and displays a direct antiproliferative activity on several cancer cell lines, whereby the LH and FSH releasing potency is extremely reduced [32]. Therefore, GnRH-III and its derivatives are considered selective and efficacious targeting moieties and represent an excellent starting point for the design of potential homing devices for targeted tumor therapy. In the past decade, a variety of GnRH-III-Dau conjugates containing an oxime bond have been developed in our laboratories, whereby Dau was attached to an incorporated aminooxyacetyl moiety (Aoa) and ^8^Lys was mainly used as the conjugation site. To improve the antitumor activity and other biochemical properties of the compounds, many different strategies have been followed including the insertion of cathepsin B labile peptide linkers or oligoethylene glycol-based spacer as well as the replacement of ^4^Ser by an acylated lysine or dimeric GnRH-III conjugates [29,33,34,35,36]. One of our most potent and efficient bioconjugates is GnRH-III-[^4^Lys(Bu), ^8^Lys(Dau=Aoa)] (**K2**), in which the Ser in position four was replaced by a butyrylated lysine [34]. Recent studies demonstrated that our lead compound **K2** possesses an enhanced stability in the presence of gastrointestinal enzymes in comparison to the ^4^Ser containing counterpart (**K1**) as well as an increased in vitro and in vivo antitumor activity [34,37].

The aim of the present work was to achieve an improved inhibitory effect on the growth of cancer cells by incorporating various unnatural amino acids into the GnRH-III sequence. Based on previously reported antiproliferative activity studies of drug-free GnRH-III derivatives [38], we synthesized and characterized a series of novel GnRH-III-Dau conjugates. Hereby, Cordopatis and coworkers have been reported that a variety of amino acid substitutions lead to an increased antiproliferative activity of GnRH-III derivatives on GnRH-R positive prostate cancer cell lines [38]. 

Here we report on the synthesis of 14 novel GnRH-III-[^8^Lys(Dau=Aoa)] conjugates with modified peptide sequences. Next to the influence of amino acid substitutions in position 3 and 7 by d-amino acids, the effect of changing the C-terminal region and the presence of a negative charge in position six was studied. For a better comparison and to ensure that no cross interactions are caused, all selected changes were performed for two different groups of compounds, one group with the natural serine in position four and a second group where the serine was exchanged by butyrylated lysine. All synthesized conjugates were analyzed for their growth inhibitory effect on GnRH-R expressing MCF-7 human breast cancer and HT-29 human colon cancer cells. The best candidate was chosen for a detailed biochemical evaluation including stability in human plasma, lysosomal degradation in the presence of rat liver lysosomal homogenate, cellular uptake by flow cytometry and confocal laser scanning microscopy (CLSM) as well as receptor binding affinity.

## 2. Materials and Methods 

### 2.1. Chemical Reagents

Amino acid derivatives, K-Oxyma Pure^®^ and Rink-Amide MBHA resin were obtained from Iris Biotech GmBH (Marktredwitz, Germany), Novabiochem^®^/Merck-Millipore (Darmstadt, Germany) and Bachem (Bubendorf, Switzerland). Boc-aminooxyacetic acid (Boc-Aoa–OH), aminooxyacetic acid, scavengers, coupling agents (1-hydroxybenzotriazole hydrate (HOBt), *N,N*′-diisopropylcarbodiimide (DIC)), and cleavage reagents (triisopropylsilane (TIS), piperidine, 1,8-diazabicyclo(5.4.0)undec-7-ene (DBU), trifluoroacetic acid (TFA), diisopropylethylamine (DIPEA), acetic anhydride (Ac_2_O), methanol (MeOH), *n*-butyric anhydride and solvent for HPLC acetonitrile (ACN) were purchased from Sigma-Aldrich Kft (Budapest, Hungary). Daunorubicin hydrochloride was provided from IVAX (Budapest, Hungary). *N,N*-Dimethylformamide (DMF), dichloromethane (DCM) and diethyl ether (Et_2_O) were purchased from Molar Chemicals Kft (Budapest, Hungary). All reagents and solvents were of analytical grade or highest available purity.

### 2.2. Synthesis of Oxime Linked GnRH-III-[^8^Lys(Dau=Aoa)] Bioconjugates

The 14 Dau-GnRH-III conjugates were prepared manually by solid phase peptide synthesis (SPPS) according to Fmoc/tBu chemistry on a Rink-Amide MBHA resin (0.73 mmol/g coupling capacity) or on a Fmoc-Ethyl-Indole AM resin (0.94 mmol/g coupling capacity). Afterwards, Dau was conjugated to the peptides in solution via oxime bond formation. For the peptide synthesis, the listed Fmoc-protected amino acid derivatives were used: Fmoc-Gly–OH, Fmoc-Pro–OH, Fmoc-Lys(Mtt)-OH, Fmoc-Lys(Dde)–OH, Fmoc-Trp-OH, Fmoc-d-Trp–OH, Fmoc-Asp(OtBu)–OH, Fmoc-Asp(OMe)–OH, Fmoc-His(Trt)–OH, Fmoc-Ser(tBu)–OH, Fmoc-d-Tic–OH. Pyroglutamic acid (Glp or <E) was coupled to the N-terminus of the peptides without any protection. The derivatives were synthesized by the following protocol, initially, the resin was washed with DMF (4 × 1 min), followed by Fmoc deprotection with 2% piperidine, 2% DBU in DMF (4 times; 2 + 2 + 5 + 10 min). For the coupling reaction 3 eq of α-Fmoc-protected amino acid derivative, 3 eq DIC and 3 eq HOBt in DMF were used (60 min). To ensure that the coupling was successful, the resin was washed with DMF (3 × 1 min) and DCM (2 × 1 min), and a ninhydrin test was performed. After peptide chain elongation, the Dde group of ^4^Lys was cleaved with 2% hydrazine in DMF (12 × 5 min) and the resin-bound peptide was washed with DMF (5 × 1 min). In the next step, the butyrylation of the ε-NH_2_ amino group was performed with 3 eq butyric anhydride and 3 eq DIPEA in DMF (2 h). Afterwards, the Mtt group of ^8^Lys was removed by using 2% TFA in DCM (6 × 5 min). The peptidyl resin was neutralized with 10% DIPEA in DCM (3 × 5 min) and Boc-Aoa-OH was coupled with DIC and HOBt (3 eq each) for 2 h. The deprotection of the side chain protecting groups and simultaneous cleavage of the peptide from the resin, was carried out with 95% TFA, 2.5% TIS and 2.5% water (*v*/*v*/*v*) in the presence of 10 eq free aminooxyacetic acid as “carbonyl capture” reagent (2 h, at room temperature (RT)) [39]. Peptides were precipitated with ice-cold Et_2_O, centrifuged, washed 3 times, dissolved in water-ACN (0.1% TFA) 4:1 (*v*/*v*) and lyophilized. After purification of the crude peptides by RP-HPLC, the eluent was evaporated and Dau was ligated to the aminooxyacetylated ^8^Lys. To perform the oxime bond formation, 10 mg/mL peptide and 1.3 eq Dau were dissolved in 0.2 M NH_4_OAc buffer (pH 5.0) and stirred overnight at RT [28]. After purification by RP-HPLC, the resulting GnRH-III bioconjugates were freeze-dried and characterized by analytical RP-HPLC and ESI-MS.

### 2.3. RP-HPLC

For the purification of the crude peptides and the bioconjugates, a KNAUER 2501 HPLC system (H. Knauer, Bad Homburg, Germany) was used either with a preparative Phenomenex Luna^®^ C18(2) column (100 Å, 10 µm, 250 mm × 21.2 mm) or with a semipreparative Phenomenex Jupiter^®^ C4 column (300 Å, 10 µm, 250 mm × 10 mm) (Torrance, CA, USA). Linear gradient elution (0 min 20% B; 5 min 20% B; 50 min 80% B) with eluent A (0.1% TFA in water) and eluent B (0.1% TFA in ACN/H_2_O (80:20, *v*/*v*)) used at a flow rate of 15 mL/min for preparative and 4 mL for semipreparative HPLC. Peaks were detected at 220 or 280 nm.

A KNAUER 2501 HPLC system was used to prove the purity of the compounds. As a stationary phase, either a Phenomenex Luna C18 column (100 Å, 5 µm, 250 mm × 4.6 mm) or a Vydac 214TP5 C4 column (300 Å, 5 µm, 250 mm × 4.6 mm) was used. A linear gradient elution (0 min 0% B; 5 min 0% B; 40 min 90%) was used at a flow rate of 1 mL/min with the eluents described above. Peaks were detected at 220 nm.

### 2.4. Mass Spectrometry

Electrospray ionization (ESI) mass spectrometric analyses were performed on an Esquire 3000+ ion trap mass spectrometer (Bruker Daltonics, Bremen, Germany). Spectra were acquired in the 50–2500 *m*/*z* range. Samples were dissolved in a mixture of ACN/water (1:1, *v*/*v*) and 0.1% formic acid.

For liquid chromatography-mass spectrometry (LC-MS) the same ESI mass spectrometer was used with an Agilent 1100 HPLC system and a diode array detector (Agilent, Waldbronn, Germany). A Supelco C18 column (150 mm × 2.1 mm, 3 µm) (Hesperia, CA, USA) was used with a linear gradient from 2–70% B in 25 min (eluent A: ddH_2_O, 0.1% HCOOH; eluent B: 80% ACN, 0.1% HCOOH at a flow rate of 0.2 mL/min) to separate the peptides. Spectra were recorded in positive ion mode in the 100–2500 *m*/*z* range.

### 2.5. Degradation of GnRH-III Bioconjugates in Rat Liver Lysosomal Homogenate

The rat liver lysosomal homogenate was prepared as previously described [29]. A Qubit Protein Assay Kit (ThermoFisher Scientific, Waltham, MA, USA) was used according to the manufacturer’s protocol to determine the protein concentration. The bioconjugates were dissolved in ddH_2_O to a concentration of 5 µg/µL. For the lysosomal digestion, an identical concentration of bioconjugate and rat liver lysosomal homogenate (0.25 µg/µL) was incubated in 0.2 M NH_4_OAc buffer (pH 5) at 37 °C. Aliquots of 15 µL were taken at 5 min, 1, 2, 4, 8 and 24 h and quenched with 2 µL of acetic acid. The analysis of the samples was performed by LC-MS.

### 2.6. Stability in Human Plasma

GnRH-III-(Dau) conjugates were dissolved in ddH_2_O and diluted with human plasma (90%) to a final concentration of 10 µM. The mixture was incubated at 37 °C and aliquots were taken after 0.5, 1, 2, 4, 8 and 24 h. The reaction was quenched by adding 10 µL of acetic acid. Large human plasma proteins were removed using ultra centrifugal filters with a cut-off of 10 kDa (Merck Millipore, Darmstadt, Germany). The lower molecular weight fraction was analyzed by LC-MS. Two control measurements have been performed in the same manner (1. 90% plasma plus 10% ddH_2_O; 2. 10 µM of bioconjugate in 100% ddH_2_O).

### 2.7. Cell Culturing

Dulbecco’s Modified Eagle Medium (DMEM, Lonza, Basel, Switzerland), supplemented with 10% (*v*/*v*) Fetal Bovine Serum (FBS, Lonza), l-glutamine (2 mM, Lonza), non-essential amino acids (NEAA, Sigma-Aldrich Kft), sodium pyruvate (1 mM, Lonza) and Penicillin-Streptomycin (Lonza) was used to culture MCF-7 human breast adenocarcinoma cells (ATCC:HTB-22). HT-29 human colon adenocarcinoma cells (ATCC:HTB-38) were maintained in RPMI-1640 (Lonza), supplied with 10% FBS, l-glutamine and Penicillin-Streptomycin. MDA-MB-231 breast adenocarcinoma cells were cultured in DMEM (Lonza), supplemented with 10% FBS and Penicillin-Streptomycin. Cells were maintained in plastic culture dishes at 37 °C with a humidified atmosphere containing 5% CO_2_/95% air.

### 2.8. In Vitro Cytostatic Effect 

5 × 10^3^ cells per well were seeded to 96-well plates (Sarstedt, Nümbrecht, Germany), in a complete cell medium. After 24 h, the cells were treated with bioconjugates in a serum-free medium (concentration range 0.068–150 µM, control wells were treated with a serum-free medium). On the next day, cells were washed two times with a serum-free medium and incubated in a complete medium for 48 h. The cell viability was determined using alamarBlue reagent^®^ (ThermoFisher Scientific) by following the manufacturer’s instructions. A Synergy H4 multi-mode microplate reader (BioTek, Winooski, VT, USA) was used for fluorescence detection (λ_Ex_ = 570 and at λ_EM_ = 620 nm). Experiments were performed twice, using four parallels per concentration. Cell viabilities (and IC_50_ values) were calculated with Origin Pro8 (OriginLab Corp., Northampton, MA, USA.) using a nonlinear regression (sigmoidal dose–response).

### 2.9. Cellular Uptake Determination by Flow Cytometry

The cellular uptake of the bioconjugates was studied on MCF-7 and HT-29 cells. Cells were seeded (10^5^ cells/well) in a complete cell medium to 24-well plates (Sarstedt). On the next day, the cells were treated with the conjugates (3.125 to 50 µM) in a serum-free medium After 6 h, cells were washed with HPMI medium (100 mM NaCl, 5.4 mM KCl, 0.4 mM MgCl_2_, 0.04 mM CaCl_2_, 10 mM Hepes, 20 mM glucose, 24 mM NaHCO_3_ and 5 mM Na_2_HPO_4_ at pH 7.4) and detached with trypsin-EDTA (10 min, 37 °C). Trypsinization was stopped by HPMI/10% FBS and cells were centrifuged at 216× *g* for 5 min at 4 °C. Afterwards, the supernatant was removed and the cells were resuspended in HPMI medium. To detect the intracellular fluorescence intensity (that is proportional to the cellular uptake), samples were measured by a BD LSR II flow cytometer (BD Bioscience, Franklin Lakes, NJ, USA) and the obtained data were analyzed by FACSDiVa (BD Bioscience) 5.0 software.

### 2.10. Confocal Microscopy Imaging

MCF-7 cells were seeded in a complete cell medium to 24-well plates, which contained cover glasses (thickness 1, Assistant, Karl Hecht GmbH & Co KG, Sondheim/Rhön, Germany). After one day, the treatment was performed in a serum-free medium for different time points (5, 15, 30 and 60 s as well as 5, 10, 30 and 60 min). Afterwards, cells were washed twice with phosphate buffered saline (PBS) and fixed by 4% paraformaldehyde for 20 min at 37 °C. To stain the nuclei, the samples were washed three times with PBS and incubated for 15 min with 4′,6-diamidine-2-phenylindole dihydrochloride (DAPI, 0.2 µg/mL, dissolved in PBS, Sigma-Aldrich Kft). After washing, cover glasses were mounted to microscopy slides (VWR International, Debrecen, Hungary) by Mowiol^®^ 4–88 mounting medium (Sigma-Aldrich Kft). Confocal microscopy studies were performed on a Zeiss LSM 710 system (Carl Zeiss Microscopy GmbH, Jena, Germany) with a 40X oil objective and ZEN Lite (Carl Zeiss Microscopy GmbH) software was used for image processing.

### 2.11. Western Blotting

GnRH-receptor expression was determined by western blot analysis of whole cell lysates. Cells (10^6^ cells/well) were seeded one day in advance to six-well plates (Sarstedt) in duplicates. Before harvesting, the cells were washed two times with PBS and lysed in lysis buffer (50 mM Tris pH 7.4, 150 mM NaCl, 2 mM EDTA, 1% Triton-X 100 and Protease Inhibitor Cocktail (Halt)). Samples were incubated for 30 min on ice followed by 30 min centrifugation at 16,000× *g*. Total protein quantity of the supernatant was measured with the Qubit Protein Assay Kit (Thermo Fisher Scientific). Subsequently, cell lysates containing an equal amount of total protein were loaded to 10% Tris-tricine gel and then blotted to a PVDF membrane (Merck Millipore). To detect the GnRHR receptor, an anti-GnRH-R antibody was used (Proteintech, Rosemant, IL, USA, Catalog Number:19950-1AP, produced in rabbit, 1:1000) followed by incubation with an anti-rabbit-HRP secondary antibody (Santa Cruz Biotechnology, Dallas, TX, USA, sc-2004, produced in goat, 1:3000). Chemiluminescence was detected by ECL Substrate (Western Lightning Plus-ECL, PerkinElmer, Waltham, MA, USA). As a loading control, actin was detected (after stripping of the membrane) by an anti-actin primary antibody (Santa Cruz Biotechnology, sc-1616, produced in goat, 1:2000) and anti-goat-HRP secondary antibody (Santa Cruz Biotechnology, sc-2354, produced in mouse, 1:3000).

### 2.12. Radioligand Binding Studies

Ligand competition assays with radiolabeled triptorelin were performed to evaluate the binding affinity of **K2** and **10** to GnRH-RI expressed on human pituitary and human prostate cancer cells as reported earlier [34,40,41,42]. Tissue samples of human prostate cancer cells were obtained from a patient at the time of initial surgical treatment and normal human pituitary tissue (anterior lobe) derived by autopsy. All subjects gave their informed consent for inclusion before they participated in the study. The collection and the use of these specimens for our studies were conducted in accordance with the Declaration of Helsinki and approved by the local Institutional Ethics Committee (UD REC/IEC 4831-2017). To study the binding affinity of the compounds, cell membrane homogenates were prepared as previously described [34,40,41,43]. Apart from that, triptorelin was radioiodinated using the chloramine-T method, followed by purification by RP-HPLC [34,40,41,44]. To determine the binding affinities of the nonradio-labeled GnRH-III bioconjugates to GnRH-RI, the displacement of [^125^I]-GnRH-I-[^6^d-Trp] was studied by an in vitro ligand competition assay [34,40,41,42]. Hence, membrane homogenates which contained 50–160 mg protein were incubated in duplicate or triplicate with 60–80,000 cpm [^125^I]-GnRH-I-[^6^d-Trp] and increasing concentration (1 pM–1 µM) of nonradioactive bioconjugates as competitive binders in 150 mL binding buffer. To determine the protein concentration by the method of Bradford, a Bio-Rad protein assay kit (Bio-Rad Laboratories, USA) was used. The LIGAND-PC computerized curve-fitting program of Munson and Rodbard was used to determine the receptor binding characteristics and IC_50_ values [34,40,41,42]. 

### 2.13. Statistical Analysis

Due to the non-normal distribution (based on the performed Shapiro-Wilk normality tests), Wilcoxon paired-sample tests were used to compare the effects of **K2** and **10** on cell viability and on cellular uptake [45]. A *p*-value < 0.05 was considered as significant. The statistical analyses were performed using R version 3.4.1. [46].

## 3. Results

### 3.1. Synthesis and Characterization of Oxime Bond-Linked GnRH-III-Dau Bioconjugates

**Scheme 1:**
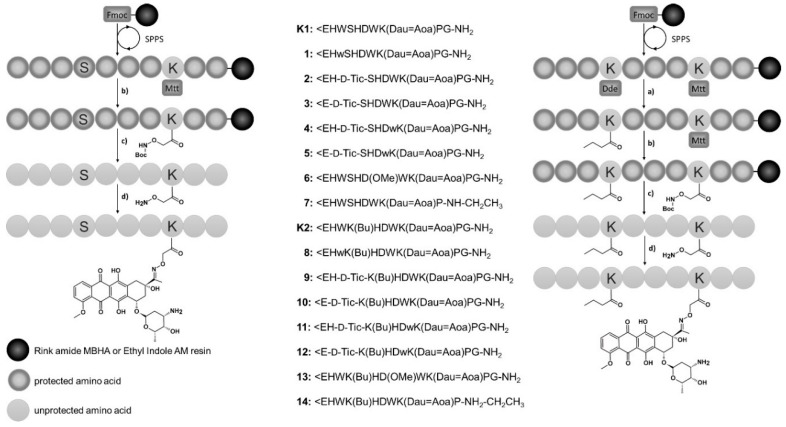
Synthesis of GnRH-III- Dau conjugates. (**a**) (1) 2% hydrazine in DMF 12 × 5 min; (2) 3 eq butyric anhydride, 3 eq DIPEA in DMF, 2 h. (**b**) (1) 2% TFA in DCM, 6 × 5 min; (2) 10% DIPEA in DCM, 3 × 5 min; (3) 3 eq Boc-Aoa-OH, 3 eq HOBt, 3 eq DIC in DMF, 1 h. (**c**) 95% TFA, 2.5% TIS, 2.5% H_2_O, 10 eq H-Aoa-OH, 2h. (**d**) 1.3 eq Dau in 0.1 M NH4OAc buffer (pH 5), overnight. Fmoc: 9-fluorenylmethoxycarbonyl, SPPS: solid phase peptide synthesis, Dde: 1-(4,4-dimethyl-2,6-dioxocyclohex-1-ylidene)ethyl, Mtt: 4-methyltrityl, Bu: butyryl; Aoa: aminooxyacetyl, Dau: daunorubicin.

The GnRH-III-Dau conjugates were prepared by standard Fmoc-SPPS using orthogonal lysine protecting groups (Scheme 1). Except for compound **13** (Supplementary Material Figure S1), Fmoc-Ser(tBu)-OH or Fmoc-Lys(Dde)-OH was incorporated in position four and Fmoc-Lys(Mtt)-OH in position eight. The Dde group was removed after peptide assembly followed by butyrylation of the ^4^Lys side chain with butyric anhydride. Afterwards, the Mtt group was cleaved under mild acidic conditions and Boc-Aoa-OH was coupled to the ε-amino group of ^8^Lys. All GnRH-III peptides were cleaved from the resin with an appropriate TFA-scavenger mixture and purified by reverse-phase HPLC. The conjugation of Dau to the aminooxyacetyl moiety was carried out in solution by formation of an oxime bond [28]. All GnRH-III-Dau conjugates were purified again by preparative HPLC. To prove the high purity (≥95%) of the final products and to ensure the absence of free Dau, the bioconjugates were characterized by analytical HPLC using two different column types (C4 and C18) and mass spectrometry (Table 1, Supplementary Material Figures S2–S17). 

### 3.2. In Vitro Cytostatic Effect of the Bioconjugates

Initially, GnRH-R expression was verified on HT-29, MCF-7 and MDA-MB-231 human cancer cells by western blot analysis. All three cell lines display a band at approximately 38 kDa, which can be considered as the full-length human GnRH-R (Supporting Information S18) [47]. Apart from that, we detected additional bands with higher molecular weights (55–70 kDa), which is in accordance with reported findings and can be assumed to be glycosylated forms of GnRH-Rs [48,49,50]. 

**Figure 1:**
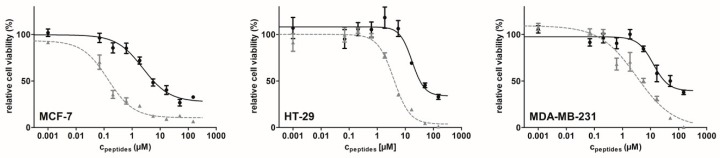
Cytostatic effect of the GnRH-III conjugate **K2** (●) and **10** (▲) on MCF-7 (**left**), HT-29 (**middle**) and MDA-MB-231 (**right**) human cancer cells after 72 h (24 h treatment and an additional 48 h incubation). Curves obtained by non-linear regression (sigmoidal dose response, error bars represent the standard deviation of four parallels, the measurements were repeated twice).

In order to investigate the influence of sequence modification on the in vitro cancer cell viability of the novel GnRH-III-Dau bioconjugates, a resazurin based cell viability assay was performed. The in vitro cytostatic effect was initially determined on estrogen-receptor α positive (ERα+) breast adenocarcinoma cancer cells (MCF-7) and on colon carcinoma cells (HT-29). To ensure the comparability to previous results, the well-studied GnRH-III bioconjugates GnRH-III-[^8^Lys(Dau=Aoa)] (**K1**) and GnRH-III-[^4^Lys(Bu), ^8^Lys(Dau=Aoa)] (**K2**) were used as internal standards and positive controls [34,35]. All GnRH-III-Dau conjugates inhibited cell proliferation in a dose-dependent manner on MCF-7 as well as on HT-29 human cancer cells (Table 2). We obtained IC_50_ values in the low micromolar range on MCF-7 cells varying between 0.14 and 6.64 µM. In the case of HT-29 colon cancer cells, the determined IC_50_ values were slightly higher and within a range of 3.31–19.10 µM. With exception of compound **10**, no significant difference of the cancer cell growth inhibitory effect of the novel bioconjugates and the control conjugates (**K1** and **K2**) could be detected. However, the replacement of ^3^Trp by ^3^d-Tic in connection with the deletion of ^2^His led to an increased cytostatic effect of **10** on both of the analyzed cell lines. The IC_50_ value of bioconjugate **10** was more than 15-times lower on ERα+ breast cancer MCF-7 cells and 5-times lower on colon cancer cells HT-29 compared to the control compound **K2**. Based on these promising findings, the growth inhibitory effects of conjugate **10** and the related ^2^ΔHis-^3^d-Tic (**3**, **5** and **12**) conjugates, as well as the ^10^ΔGly-NH-Et (**7** and **14**) containing compounds, were studied on estrogen receptor negative (ERα-) MDA-MB-231 breast cancer cells. The corresponding IC_50_ values are shown in Table 2. The GnRH-III-Dau conjugate **10** revealed also on this cell line the highest anticancer activity with an IC_50_ value of 2.49 µM. The comparison of the dose-dependent growth inhibitory effect of **10** and **K2** on the three cancer cell lines is shown in Figure 1. Considering the results of all three cell lines, only compound **10** which contains the N-terminal modification ^2^ΔHis-d-Tic-Lys(Bu), displayed clearly a reduced cell viability, while the conjugates bearing other substitutions yielded IC_50_ values which vary only slightly in comparison to the controls. The enhancement of the cytostatic effect might be a result of an improved cellular uptake and/or release of the active metabolite in lysosomes. For a better understanding of our findings, we decided to perform further studies of candidate **10** in direct comparison with our lead compound **K2**. 

### 3.3. Stability in Human Plasma

The stability of peptide-drug conjugates in human blood plasma is of great importance for the therapeutic application. Therefore, the stability of our two lead compounds **K2** and **10** was determined in 90% human plasma and detected by LC-MS. Both GnRH-III-Dau conjugates were stable in human plasma for at least 24 h at 37 °C, which is of great importance for upcoming in vivo studies (Supplementary Figure S18).

### 3.4. Degradation of GnRH-III Bioconjugates in Rat Liver Lysosomal Homogenate

**Figure 2:**
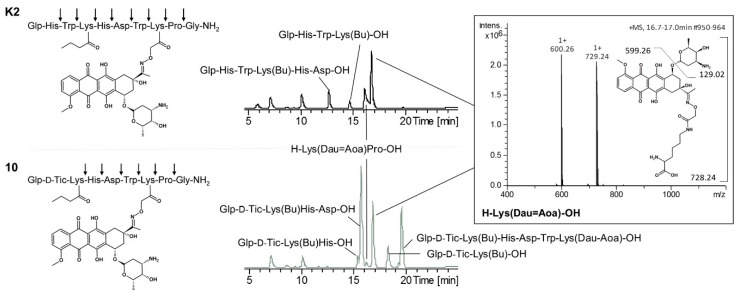
Degradation of the GnRH-III conjugates **K2** and **10** in presence of lysosomal rat liver homogenate. Cleavage sites produced by lysosomal enzymes are shown by full line arrows (**left**). LC chromatograms of **K2** and **10** after 24 h degradation (**middle**) and MS spectra of the active metabolite H-Lys(Dau=Aoa)-OH (**right**).

To enable the selective delivery of the drug to cancer cells, not only the stability under physiological conditions is of high relevance but also the release of the drug or active drug metabolites at the tumor tissue. Therefore, we determined the release of the smallest Dau-containing metabolite in presence of rat liver lysosomal homogenate. The degradation of the conjugates **K2** and **10** and thereby the formation of the smallest Dau-containing metabolite was followed by LC-MS at different time points. Due to the high stability of the oxime bond, the free drug could not be detected. Both conjugates were degraded resulting in various peptide fragments (Figure 2 and Supporting Information Table S1). In general, **K2** displayed a higher lability in the presence of the lysosomal enzymes than **10**, especially the N-terminal region of **K2** was less stable. Nevertheless, the presence of the smallest Dau metabolite H-Lys(Dau=Aoa)-OH could already be detected after 1 h of incubation for both GnRH-III-Dau derivatives, which is of high relevance for the biological activity of the conjugate.

### 3.5. Cellular Uptake Determination by Flow Cytometry

**Figure 3:**
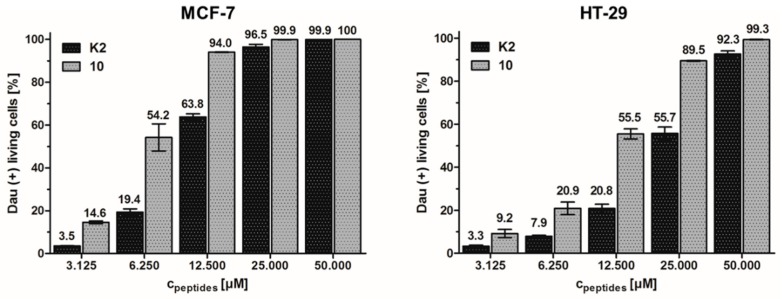
Cellular uptake of the GnRH-III conjugates **K2** and **10** on MCF-7 (**left**) and HT-29 (**right**) cancer cells after 6 h treatment determined by flow cytometry. The cellular uptake of **10** was significantly higher than the uptake of conjugate **K2** for both cells (paired Wilcoxon test, *p* = 0.004 and *p* = 0.005, respectively).

To compare the cellular uptake of **K2** and **10**, MCF-7 and HT-29 cells were treated with different concentrations of compounds and after 6 h incubation, flow cytometry studies were carried out. Hereby, only living cells have been considered revealing that compound **10** was taken up more effectively than **K2** in both cancer cell lines (Figure 3). In the case of MCF-7 cells, the uptake was at the lowest compound concentration (3.125 µM) already 4 times higher for **10** (14.55%) than for **K2** (3.5%). At 6.25 µM concentration, 54.2% (**10**) and 19.35% (**K2**) of living cells were Dau positive, while at 12.5 µM compound concentration, 94% (**10**) and 63.8% (**K2**) uptake could be detected. At the two highest concentrations (25 and 50 µM), the cellular uptake on MCF-7 cells was between 96.45 and 100% for both conjugates. On HT-29 colon cancer cells, bioconjugate **10** (3.125 µM) was taken up by 9.15% of the cells whereby the uptake of **K2** was 3.25%. This tendency remains similar for the other concentrations, while at the highest concentration (50 µM), a cellular uptake of 99.3% was detected for compound **10** and 92.5% for **K2**. Considering these results, we assume that the improved cytostatic effect is mainly related to the improved cellular uptake of compound **10**. 

### 3.6. Cellular Uptake Determination by Confocal Microscopy Imaging

**Figure 4:**
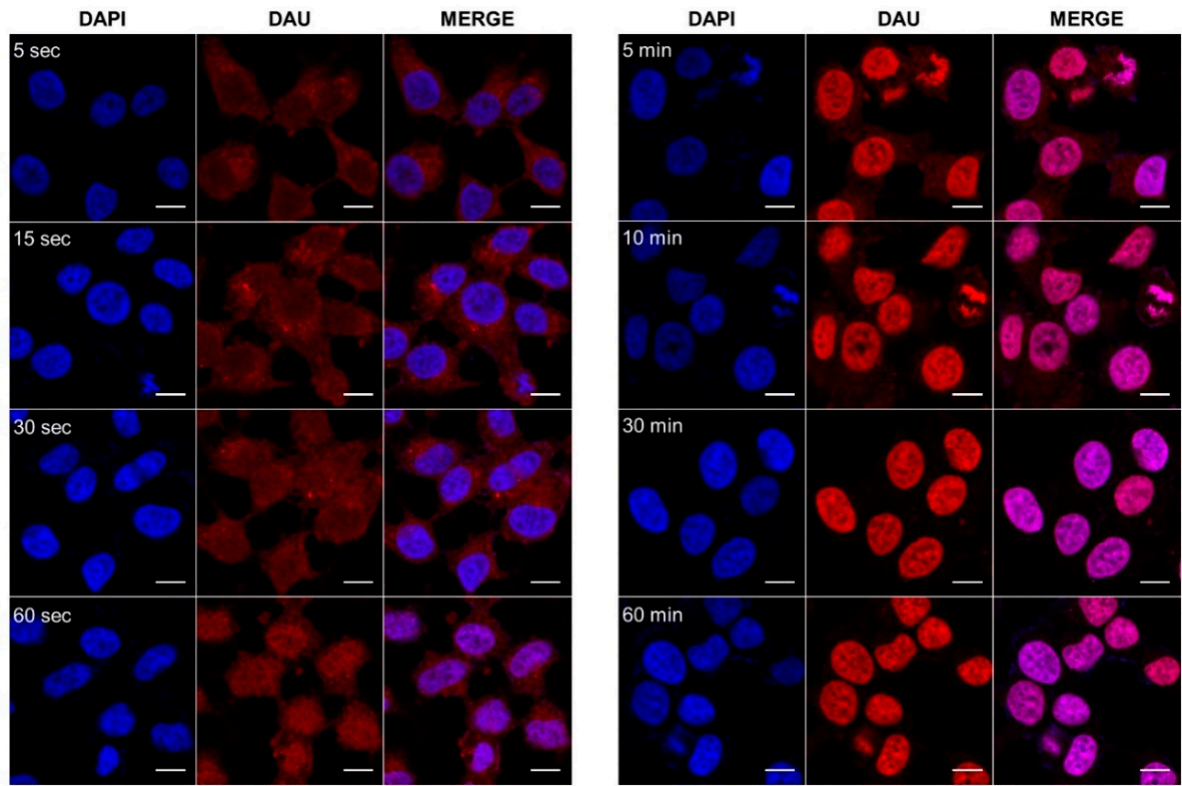
Time-dependent cellular uptake of GnRH-III conjugate **10** (DAU) on MCF-7 breast cancer cells. Cells were plated onto glass cover slips as described in Section 2.10. Fluorescent pictures were taken using a confocal laser scanning microscope (after 5, 15, 30 and 60 s (**left**) as well as 5, 10, 30 and 60 min (**right**) incubation). DAPI (blue) and GnRH-III conjugate **10** (DAU, red) were excited at 405 and 458 nm respectively. Emission was collected at 450–510 nm for DAPI, 550–599 nm for GnRH-III conjugate **10** (DAU). The scale bar represents 10 µm.

In order to visualize the cellular uptake and the localization of compound **10** in MCF-7 breast cancer cells, confocal fluorescence microscopy studies were performed. The cells were incubated with the conjugate (40 µM) for different time intervals from five seconds up to one hour, followed by fixation and preparation for imaging. DAPI-staining was performed to verify the localization of Dau and Dau-containing metabolites in the nuclei. After 5 min incubation, the Dau signal was detected mainly in the nuclei, whereby the obtained images within the first minute display the Dau signal predominantly in the cytosol and in small vesicles (Figure 4). These results indicate that the drug-conjugate could be efficiently taken up by the cancer cells and reached its site of action already within 5 min. 

### 3.7. Radioligand Binding Studies

**Figure 5:**
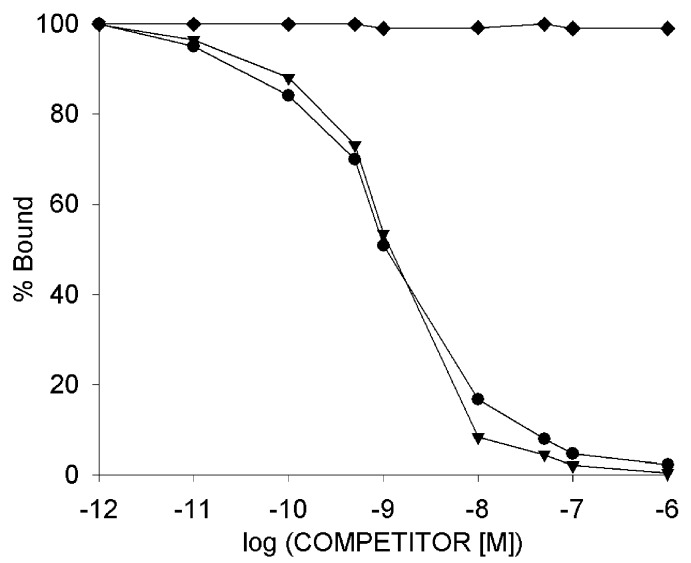
Representative displacement of [^125^I]-GnRH-I-[^6^d-Trp] binding to membrane fractions of human prostate cancer specimens by increasing concentrations of GnRH-III conjugates **10** (●) and **K2** (▼). Other unrelated peptides, like somatostatin, human growth hormone and epidermal growth factor (♦) did not displace the radioligand. Each point represents the mean of duplicate or triplicate determinations.

To ensure that the cellular uptake is related to the receptor-mediated pathway, we determined the binding affinities of bioconjugate **10** and our lead compound **K2** to GnRH-receptors by ligand competition assay. Increasing concentrations of the conjugates were used to displace radiolabeled triptorelin from GnRH-receptors expressed on human pituitaries and human prostate cancer tissues. The binding affinity of bioconjugate **10** was compared with that of our lead compound **K2**. Both conjugates displaced [^125^I]-triptorelin with high potency and their IC_50_ values were in the low nanomolar range. Both conjugates revealed a similar binding affinity for human pituitary tissues (**10**: 3.53 ± 0.96 nM and **K2**: 3.59 ± 2.17 nM) and human prostate cancer tissues (**10**: 2.79 ± 1.24 nM and **K2**: 3.43 ± 2.01 nM; Figure 5). These low nanomolar IC_50_ values of **K2** and **10** demonstrated that both conjugates have a high binding affinity to the GnRH-receptors and that the receptor binding is not influenced by the N-terminal amino acid substitution of compound **10**.

## 4. Discussion

Cancer is one of the most serious diseases worldwide and according to the WHO this malignancy was the second leading cause of death with 8.8 million deaths in 2015 [51]. Due to this, the development of efficient diagnostic and therapeutic tools is of high relevance. One promising approach for the treatment of cancer is targeted tumor therapy, which is based on the fact that receptors for many regulatory ligands are highly expressed in many tumor cells. Due to this, hormone peptides like GnRH and its derivatives are promising homing devices for the selective delivery of antitumor drugs to cancer cells. The use of GnRH derivatives for targeted tumor therapy offers valuable benefits since they possess an antiproliferative activity, which provides an additional inhibitory effect on tumor growth [52]. Furthermore, it has been reported that GnRH-Rs are not only expressed in reproductive system related tumors but also in unrelated cancer types, which extend the potential of their use as targets in cancer therapy [53].

Apart from human GnRH-I, many native GnRH isoforms have been isolated from different species showing that the N- and C-terminal regions are highly conserved and essential for receptor binding and activity [54,55]. These studies have shown that the active conformation of mammalian GnRH exhibits a β-turn structure formed by residues 5–8 whereby the N- and C-termini face each other [54,55]. However, NMR studies comparing the human GnRH-I and the sea lamprey analog GnRH-III pointed out that their conformations are substantially different. While the results of GnRH-I confirm a defined U-shape structure, GnRH-III seems to be less bent and according to the NMR spectra, an ordered extended backbone conformation can be proposed [38]. Moreover, it could be demonstrated that amino acid substitutions within the GnRH-III sequence can lead to an improved antiproliferative activity even if the replaced residues are assumed to be crucial for the biological activity [38]. 

The aim of the present study was to develop novel GnRH-III-Dau conjugates with an improved antitumor activity by incorporating various unnatural amino acids within the peptide sequence. Hereby, we paid special attention to the effect of amino acid exchange in position seven by d-Trp and the replacement of ^3^Trp by d-Trp as well as d-1,2,3,4-tetrahydroisoquinoline-3-carboxylic acid (d-Tic) and in combination with ^2^His deletion. Moreover, we investigated the influence of ^6^Asp side chain methylation and of modifying the *C*-terminus to ^10^ΔGly-NHEt. Based on our previous studies, which pointed out that an exchange of ^4^Ser by ^4^Lys(Bu) results in an enhanced in vitro and in vivo anticancer activity, we decided to investigate two sets of GnRH-III-Dau conjugates containing either the natural serine in position four or Lys(Bu). 

In the present work, we report the synthesis and biological evaluation of 14 novel oxime linked GnRH-III-Dau conjugates. After synthesis and purification, the final conjugates have been analyzed for their cytostatic effect on reproductive system related (MCF-7) and unrelated (HT-29) GnRH-R expressing cancer cells. Due to the fact that immortal cell lines can differ in their genotypic and phenotypic characteristics depending on the passage and state of confluence, we used our well-established GnRH-III-Dau derivatives **K1** and **K2** for a better comparison and as an internal standard [34,35]. All new analogs showed a significant growth inhibitory effect on both human cancer cell lines with IC_50_ values in the low micromolar range, which vary only slightly from that of the control peptides. Besides that, the conjugate GnRH-III-[^2^ΔHis, ^3^d-Tic, ^4^Lys(Bu), ^8^Lys(Dau=Aoa)] (**10**) revealed an improved inhibitory effect on the growth of both cancer cell lines, whereby the cytostatic effect on MCF-7 breast cancer cells was increased by more than one order of magnitude in comparison to **K2**. In contrast, the C-terminal modification ^10^ΔGly-NHEt (**7**, **14**) did not result in a significant change of the cell viability in comparison with the control peptides (**K1**, **K2**) on both cell lines. These results are in agreement with earlier studies of drug-free GnRH-III derivatives [56], while in case of GnRH-I derivatives, the replacement of Gly-NH_2_ with ethyl amide provides the more potent GnRH-I agonist fertirelin [57,58]. Moreover, by combining the modification ^9^Pro-NHEt with ^6^Gly replacement by d-amino acids, superagonists like buserelin or leuprolide are produced, which possess a much higher activity than the native GnRH-I or fertirelin [12,58]. It could be shown that the increased activity is mainly related to the ^6^Gly substitution since d-amino acids in position six enhance the β-turn conformation of GnRH-I agonists [55]. On the contrary, the incorporation of various ^6^d-Aaa did not result in an improved biological activity of GnRH-III-Dau conjugates [42]. Apart from that, it could be shown that the negative charge of the ^6^Asp side chain is not mandatory for the biological activity of GnRH-III, beyond that the methylation of the aspartate residue results in an enhanced cytostatic effect [38,59]. In contrast, the methylation of GnRH-III-Dau conjugates (**6**, **13**) could not cause a significant change of the cytostatic effect; although our results confirm that the acidic character of ^6^Asp is not essential for the activity of the conjugates.

According to Sealfon, the residues ^2^His and ^3^Trp might play an important role in receptor activation and biological activity of GnRH-I derivatives [54]. On the other hand, it has been reported that an exchange of ^3^Trp with d-Trp and d-Tic reduces the receptor binding affinity of GnRH-I analogs but provides an increased antiproliferative activity on MCF-7 cells [60]. However, our results of the GnRH-III-Dau conjugates, which have been modified in the ^2^His-^3^Trp and/or ^7^Trp positions revealed no substantial deterioration of the antitumor activities, which is in line with previous studies [38]. Initially, we analyzed the influence of replacing ^3^Trp by d-Trp and d-Tic. The results displayed a slightly improved biological activity of the d-Tic conjugates **2** and **9** on HT-29 cells. In the next step, we studied the influence of ^2^His deletion and/or ^7^d-Trp substitution in combination with ^3^d-Tic. On all three cell lines, the strongest positive impact on the biological activity could be observed for conjugate **10** with the N-terminal modifications ^2^ΔHis-^3^d-Tic-^4^Lys(Bu). In order to interpret these outcomes, we decided to perform further studies of candidate **10** in direct comparison with our lead compound **K2**. 

To ensure the stability of the conjugates under physiological conditions, we analyzed their durability in human blood plasma revealing that both conjugates are stable for at least 24 h, which is in accordance with our previous results of related GnRH-III-Dau derivatives [35,61]. Next to compound stability in circulation, the release of the drug within cancer cells is of high relevance. Due to the high chemical and enzymatic stability of the oxime bond, we could not detect the release of the free Dau by lysosomal enzymes, which is in agreement with prior results [34,35,42]. Nevertheless, DNA-binding studies could prove that also small Dau-containing metabolites can intercalate in DNA and thereby inhibit topoisomerase II activity, which leads to a substantial reduction of cell proliferation [29]. The degradation profile of **10** and **K2** in presence of lysosomal enzymes shows that the N-terminal part Glp-d-Tic-Lys(Bu)-His-Asp of **10** is more resistant to lysosomal enzymes. In accordance with our recent studies, already after 5 min of incubation, we could detect the fragments <EHWK(Bu)-OH and H-HDWK(Dau=Aoa))PG-NH_2_ for **K2** giving a clear evidence of the endopeptidase activity [42]. In spite of the enhanced stability of **10**, the smallest Dau-containing metabolite H-Lys(Dau=Aoa)-OH could be detected within the first hour for both conjugates. Due to the detected fragments, we assume that the active metabolite H-Lys(Dau=Aoa)-OH is rapidly released by lysosomal enzymes with carboxymono- and dipeptidase activity resulting in an efficient biological activity of both conjugates.

Since anthracyclines like Dau are highly fluorescent molecules, the cellular uptake of GnRH-III-Dau conjugates can be studied by fluorescence-activated cell sorting (FACS) and/or confocal laser scanning microscopy (CLSM) without changing the inherent properties of the compounds [62]. Thus, the uptake rate of **K2** and **10** on HT-29 and MCF-7 human cancer cells was investigated by flow cytometry. The obtained results display an increased cellular uptake for the new conjugate **10** on both cell lines. Especially, the uptake rates at low compound concentrations are considerably improved in comparison to **K2** indicating that the enhanced biological activity of **10** is mainly related to the improved cellular uptake. Besides the quantitative uptake studies by flow cytometry, we examined the subcellular disposition of compound **10** in a time-dependent manner by CLSM. In the first minute, the Dau signal was mainly detected in small cytosolic vesicles, while after five minutes the Dau-fluorescence could be predominantly identified in the nuclei. Considering that comparable study of **K2** revealed the necessity of 10 min incubation to observe the presence of Dau in nuclei, it can be assumed that the delivery of the drug to its site of action is accelerated by using bioconjugate **10** [42]. 

According to Zompra, the incorporation of ^3^d-Tic in GnRH-I derivatives can lead to a substantially reduced GnRH-receptor affinity, while the inhibitory effect on cell proliferation is increased [60]. To validate that the anticancer effect of the novel compound is still related to GnRH-receptor binding, we determined the receptor affinity of **10** and **K2** by displacement studies with radiolabeled [^125^I]-triptorelin. Based on our results both compounds bind to GnRH-receptors expressed on human pituitaries and prostate cancer tissues with high binding affinities. Moreover, the results obtained for **K2** are nicely comparable to our recent studies [42].

The low nanomolar IC_50_ values of both GnRH-III conjugates demonstrated that the binding of GnRH-I agonist triptorelin was efficiently inhibited in a competitive manner by using increasing concentrations of 1 pM to 1 µM. By taking into account that peptides unrelated to GnRH like somatostatin-14 or bombesin were not able to displace triptorelin by applying concentrations up to 1 µM, it can be assumed that the receptor-mediated uptake was not affected by the performed amino acid substitutions [40,41]. 

Considering these findings, bioconjugate **10** represents a highly promising candidate for in vivo antitumor activity studies in tumor-bearing mice. But since the tumor growth of MCF-7 cells for breast cancer xenograft depends strongly on exogenous 17β-estradiol (E2) support we analyzed the in vitro cytostatic effect of **10** also on GnRH-R positive, but ERα negative MDA-MB-231 breast cancer cells [63,64]. For a better interpretation, we include all ^2^ΔHis-^3^d-Tic containing conjugates (**3**, **5**, **10** and **11**) and the C-terminally modified GnRH-III-Dau (**7**, **14**) conjugates in this study. The obtained results revealed a similar tendency of the tumor growth inhibitory effect as we detected for MCF-7 and HT-29 cells, whereby GnRH-conjugate **10** displays a significant increase in cell growth inhibition compared to **K2**. Moreover, it has been reported that MDA-MB-231 breast cancer cells are not only ERα- but also progesterone receptor (PgR) and human epidermal growth factor receptor 2 (HER-2) negative [63,65,66]. Due to the fact that these triple negative (ERα-/PgR-/Her-2) cancer cells are considered to be very aggressive and since some commonly used chemotherapeutics like Herceptin (HER-2 targeting) lack activity on these kinds of cells, alternative approaches are necessary to affect such tumor types. Our findings support the assumption that GnRH-receptor might be a potential target to overcome these limitations since GnRH-Rs are expressed in more than 50% of human breast cancer. 

In conclusion, all 14 novel GnRH-III-Dau conjugates demonstrate an efficient cancer cell growth inhibition in vitro. Especially the conjugate GnRH-III-[^2^ΔHis,^3^d-Tic,^4^Lys(Bu),^8^Lys(Dau=Aoa)] (**10**) displays an increased cytostatic effect on all analyzed cancer cell lines in comparison to the lead compound **K2**. The detailed biochemical characterization of bioconjugate **10** indicates that this N-terminal modification leads to an improved cellular uptake of the conjugate which is accompanied by an accelerated delivery of the drug to its site of action. Receptor binding studies demonstrated the high binding affinity of our compound to GnRH-receptors expressed on human cancer tissue. Furthermore, we could ensure that the release of the active drug metabolite in lysosomes is not retarded by the d-amino acid substitutions, while the compound provides a high stability in human blood plasma which is of great importance for upcoming tumor growth inhibitory studies in vivo.

All findings of the present study demonstrate the great value of our new lead compound and indicate its high potential as a promising drug delivery system in targeted tumor therapy. In vivo studies on tumor-bearing mice are planned for the near future to further confirm its application as an efficient agent for selective cancer therapy.

## Figures and Tables

**Table 1:** Chemical characteristics of GnRH-III-Dau conjugates.

Code	[^8^Lys(Dau=Aoa)]-GnRH-III Compound	Purity [%]	RP-HPLC R_t_ [min] ^a^	ESI-MS MW_cal_/MW_exp_ [g/mol] ^b^
**K1**		≥97	21.37	1841.89/1841.66
**1**	[^3^d-Trp]	≥98	21.43	1841.89/1841.65
**2**	[^3^d-Tic]	≥98	21.22	1814.86/1814.65
**3**	[^2^ΔHis, ^3^d-Tic]	≥95	22.98	1677.72/1677.54
**4**	[^3^d-Tic, ^7^d-Trp]	>97	21.58	1814.86/1814.62
**5**	[^2^ΔHis, ^3^d-Tic, ^7^d-Trp]	≥95	23.10	1677.72/1677.53
**6**	[^6^Asp(OMe)]	≥95	21.85	1855.91/1855.64
**7**	[^10^ΔGly-NHEt]	≥97	21.18	1812.88/1812.82
**K2**	[^4^Lys(Bu)]	≥99	22.41	1953.07/1952.79
**8**	[^3^d-Trp, ^4^Lys(Bu)]	≥98	22.83	1953.07/1952.65
**9**	[^3^d-Tic, ^4^Lys(Bu)]	≥97	22.57	1926.05/1925.73
**10**	[^2^ΔHis, ^3^d-Tic, ^4^Lys(Bu)]	≥96	24.27	1788.91/1788.64
**11**	[^3^d-Tic, ^4^Lys(Bu), ^7^d-Trp]	≥96	22.77	1926.05/1925.81
**12**	[^2^ΔHis, ^3^d-Tic, ^4^Lys(Bu), ^7^d-Trp]	≥98	24.27	1788.91/1788.68
**13**	[^4^Lys(Bu), ^6^Asp(OMe)]	≥98	22.92	1967.10/1966.68
**14**	[^4^Lys(Bu), ^10^ΔGly-NHEt]	≥97	23.17	1924.07/1923.72

^a^ Column: Vydac 214TP5 C4 column (250 mm × 4.6 mm) with 5 µm silica (300 Å pore size); gradient: 0 min 0% B, 5 min 0% B, 40 min 90% B; eluents: 0.1% TFA in water (A) and 0.1% TFA in acetonitrile-water (80:20, *v*/*v*) (B); flow rate: 1 mL/min; detection at 220 nm. ^b^ Bruker Daltonics Esquire 3000+ ion trap mass spectrometer.

**Table 2:** In vitro cytostatic effects of GnRH-III-Dau conjugates on HT-29 human colon cancer, MCF-7 human and MDA-MB-231 human breast cancer cells.

Code	[^8^Lys(Dau=Aoa)]-GnRH-III Compound	HT-29 IC_50_ [µM]	MCF-7 IC_50_ [µM]	MDA-MB-231 IC_50_ [µM]
**K1**		13.89 ± 3.62	2.54 ± 0.67	8.22 ± 0.13
**1**	[^3^d-Trp]	15.25 ± 2.51	3.60 ± 0.28	n.d.
**2**	[^3^d-Tic]	8.75 ± 0.86	2.89 ± 0.62	n.d.
**3**	[^2^ΔHis, ^3^d-Tic]	10.32 ± 1.32	2.75 ± 0.17	9.35 ± 1.93
**4**	[^3^d-Tic, ^7^d-Trp]	15.34 ± 2.63	3.42 ± 0.39	n.d.
**5**	[^2^ΔHis, ^3^d-Tic, ^7^d-Trp]	10.70 ± 0.95	1.90 ± 0.58	7.88 ± 1.24
**6**	[^6^Asp(OMe)]	10.66 ± 1.76	4.81 ± 0.72	n.d.
**7**	[^10^ΔGly-NHEt]	14.18 ± 3.59	4.88 ± 0.01	14.33 ± 1.18
**K2**	[^4^Lys(Bu)]	15.93 ± 0.99	2.36 ± 0.07	9.00 ± 1.33
**8**	[^3^d-Trp, ^4^Lys(Bu)]	15.03 ± 2.51	6.64 ± 1.58	n.d.
**9**	[^3^d-Tic, ^4^Lys(Bu)]	12.73 ± 3.10	2.56 ± 0.51	n.d.
**10**	[^2^ΔHis, ^3^d-Tic, ^4^Lys(Bu)]	3.31 ± 0.90	0.14 ± 0.01	2.49 ± 0.53
**11**	[^3^d-Tic, ^4^Lys(Bu), ^7^d-Trp,]	16.83 ± 0.66	2.57 ± 0.47	n.d.
**12**	[^2^ΔHis, ^3^d-Tic, ^4^Lys(Bu), ^7^d-Trp]	16.55 ± 0.30	2.81 ± 0.04	8.18 ± 0.18
**13**	[^4^Lys(Bu), ^6^Asp(OMe)]	18.00 ± 0.13	3.44 ± 0.51	n.d.
**14**	[^4^Lys(Bu), ^10^ΔGly-NHEt]	17.84 ± 0.08	2.23 ± 0.40	12.41 ± 2.30

n.d.—not determined, all values represent mean ± SE.

## Supplementary Materials

The following are available online at http://www.mdpi.com/1999-4923/10/4/223/s1, Figure S1: Synthesis strategy for **13**, Figure S2: RP-HPLC profile and ESI-ion trap mass spectrum of **K1**, Figure S3: RP-HPLC profile and ESI-ion trap mass spectrum of **K2**, Figure S4: RP-HPLC profile and ESI-ion trap mass spectrum of **1**, Figure S5: RP-HPLC profile and ESI-ion trap mass spectrum of **2**, Figure S6: RP-HPLC profile and ESI-ion trap mass spectrum of **3**, Figure S7: RP-HPLC profile and ESI-ion trap mass spectrum of **4**, Figure S8: RP-HPLC profile and ESI-ion trap mass spectrum of **5**, Figure S9: RP-HPLC profile and ESI-ion trap mass spectrum of **6**, Figure S10: RP-HPLC profile and ESI-ion trap mass spectrum of **7**, Figure S11: RP-HPLC profile and ESI-ion trap mass spectrum of **8**, Figure S12: RP-HPLC profile and ESI-ion trap mass spectrum of **9**, Figure S13: RP-HPLC profile and ESI-ion trap mass spectrum of **10**, Figure S14: RP-HPLC profile and ESI-ion trap mass spectrum of **11**, Figure S15: RP-HPLC profile and ESI-ion trap mass spectrum of 1**2**, Figure S16: RP-HPLC profile and ESI-ion trap mass spectrum of **13**, Figure S17: RP-HPLC profile and ESI-ion trap mass spectrum of **14**, Figure S18: Western blot performed on whole cell lysates of MDA-MB-231, HT-29 and MCF-7 cancer cells, Figure S19: Stability of the bioconjugates **K2** and **10** in human plasma, Figure S20: Mean of fluorescence of the cellular uptake of the GnRH-III conjugates **K2** and **10** on MCF-7 and HT-29 cancer cells after 6 h treatment determined by flow cytometry, Figure S21: Cellular uptake of the GnRH-III conjugates **K2** and **10** on MCF-7 and HT-29 cancer cells after 6 h treatment determined by flow cytometry (FACS histograms). Table S1: Fragments of GnRH-III-Dau conjugates **K2** and **10** produced by rat liver homogenate.
