# Supplementary material for: Enhanced In Vitro Antitumor Activity of GnRH-III-Daunorubicin Bioconjugates Influenced by Sequence Modification
Source: Pharmaceutics. 2018 Nov 9;10(4):223. doi: 10.3390/pharmaceutics10040223 (PMC6320914; doi:10.3390/pharmaceutics10040223)
Supplement: Supplementary file 1 [file pharmaceutics-10-00223-s001.pdf]

pharmaceutics

Supplementary Materials: Enhanced In Vitro Antitumor Activity of GnRH-III-Daunorubicin Bioconjugates Influenced by Sequence Modification

Sabine Schuster, Beáta Biri-Kovács, Bálint Szeder, László Buday, János Gardi, Zsuzsanna Szabó, Gábor Halmos and Gábor Mező

Table of Content

Synthesis strategy of bioconjugate **13** 3

RP-HPLC profile and ESI-ion trap mass spectrum of GnRH-III-[8Lys(Dau=Aoa)] (**K1**) 4

RP-HPLC profile and ESI-ion trap mass spectrum of GnRH-III-[4Lys(Bu), 8Lys(Dau=Aoa)] (**K2**) 4

RP-HPLC profile and ESI-ion trap mass spectrum of GnRH-III-[3Trp, 8Lys(Dau=Aoa)] (**1**) 4

RP-HPLC profile and ESI-ion trap mass spectrum of GnRH-III-[3D-Tic, 8Lys(Dau=Aoa)] (**2**) 5

RP-HPLC profile and ESI-ion trap mass spectrum of GnRH-III-[2ΔHis, 3D-Tic, 8Lys(Dau=Aoa)] (**3**) 5

RP-HPLC profile and ESI-ion trap mass spectrum of GnRH-III-[3D-Tic, 7D-Trp 8Lys(Dau=Aoa)] (**4**) 5

RP-HPLC profile and ESI-ion trap mass spectrum of GnRH-III-[2ΔHis, 3D-Tic, 7D-Trp 8Lys(Dau=Aoa)] (**5**) 6

RP-HPLC profile and ESI-ion trap mass spectrum of GnRH-III-[6Asp(OMe), 8Lys(Dau=Aoa)] (**6**) 6

RP-HPLC profile and ESI-ion trap mass spectrum of GnRH-III-[8Lys(Dau=Aoa), 10ΔGly-NH-Et] (**7**) 6

RP-HPLC profile and ESI-ion trap mass spectrum of GnRH-III-[3Trp, 4Lys(Bu), 8Lys(Dau=Aoa)] (**8**) 7

RP-HPLC profile and ESI-ion trap mass spectrum of GnRH-III-[3D-Tic, 4Lys(Bu), 8Lys(Dau=Aoa)] (**9**) 7

RP-HPLC profile and ESI-ion trap mass spectrum of GnRH-III-[2ΔHis, 3D-Tic, 4Lys(Bu), 8Lys(Dau=Aoa)] (**10**) 7

RP-HPLC profile and ESI-ion trap mass spectrum of GnRH-III-[3D-Tic, 4Lys(Bu), 7D-Trp 8Lys(Dau=Aoa)] (**11**) 8

RP-HPLC profile and ESI-ion trap mass spectrum of GnRH-III-[2ΔHis, 3D-Tic, 4Lys(Bu), 7D-Trp 8Lys(Dau=Aoa)] (**12**) 8

RP-HPLC profile and ESI-ion trap mass spectrum of GnRH-III-[4Lys(Bu), 6Asp(OMe), 8Lys(Dau=Aoa)] (**13**) 8

RP-HPLC profile and ESI-ion trap mass spectrum of GnRH-III-[4Lys(Bu), 8Lys(Dau=Aoa), 10ΔGly-NH-Et] (**14**) 9

Stability of the bioconjugates **K2** and **10** in human plasm 9

Fragments of **K2** and **10** produced in the presence of rat liver homogenate 10

Cellular uptake of **K2** and **10** by flow cytometry 10

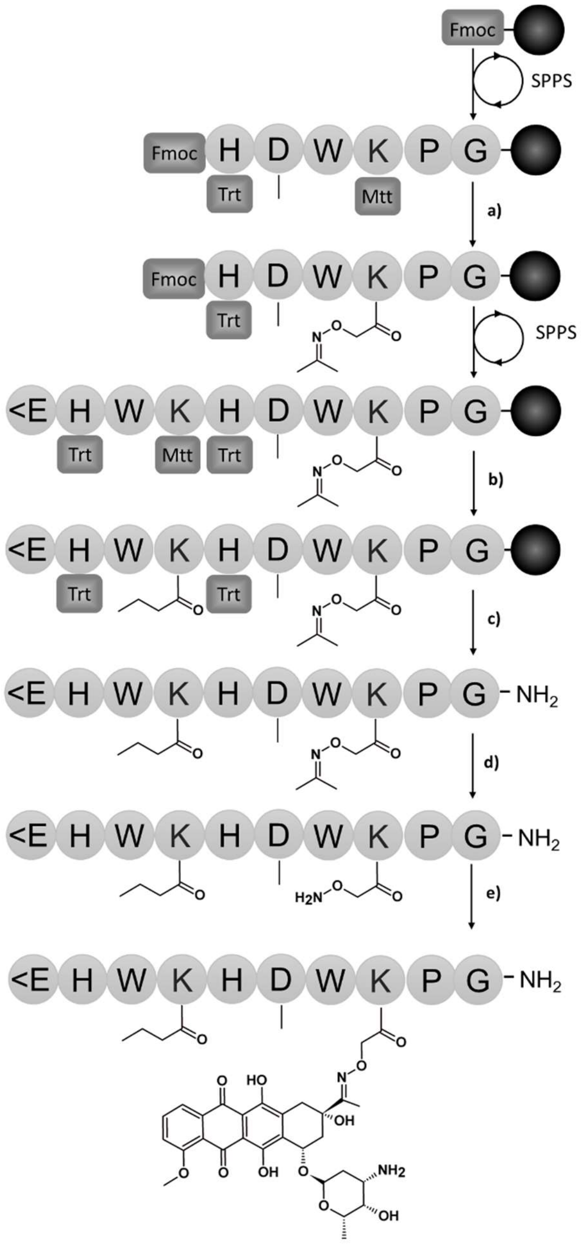

Figure S1. Synthesis strategy for **13** (<EHWK(Bu)HD(OMe)WK(Dau=Aoa)PG-NH_2. Bioconjugate **13** was prepared by manual SPPS according to Fmoc/tBu chemistry on a Rink-Amide MBHA resin (0.73 mmol/g coupling capacity). The general protocol for the synthesis started with DMF-washing (4 x 1 min), followed by Fmoc deprotection with 2% piperidine, 2% DBU in DMF (4 times; 2 + 2 + 5 +10 min). The coupling reaction was performed by using 3 eq of α-Fmoc-protected amino acid derivative, 3 eq DIC and 3 eq HOBt in DMF (60 min). After washing with DMF (3 x 1 min) and DCM (2 x 1 min) the success of the coupling was controlled by ninhydrin test.

a) (1) 2% TFA in DCM - 6x5 min; (2) 10% DIPEA in DCM - 3x5 min; (3) 10 eq >=Aoa-OH, 10 equiv K-Oxima pure ®, 10 eq DIC in DMF – 2h

b) (1) 2% TFA in DCM - 6x5 min; (2) 10% DIPEA in DCM - 3x5 min; (3) 3 eq butyric anhydride, 3 eq DIPEA in DMF – 2h

c) 95% TFA, 2.5% TIS, 2.5% H_2O – 2h

d) 2 M methoxylamine hydrochloride in 0.2 M NH_4OAc-buffer (pH 5) – 2-3h (reaction control by analytical HPLC)

e) 1.3 equiv Dau in 0.2 M NH_4OAc-buffer (pH 5) – overnight

Aoa: aminooxyacetyl, **Bu:** butyl; **Dau:** daunorubicine, **Fmoc:** 9-fluorenylmethoxycarbonyl; **Mtt:** 4-methyltrityl; **SPPS:** solid phase peptide synthesis; **tBu:** *tert*-butyl, **Trt:** trityl; **>=:** isopropylidenyl ●**:** Rink-Amide-MBHA-resin; ●**:** amino acid; ■**:** protecting group

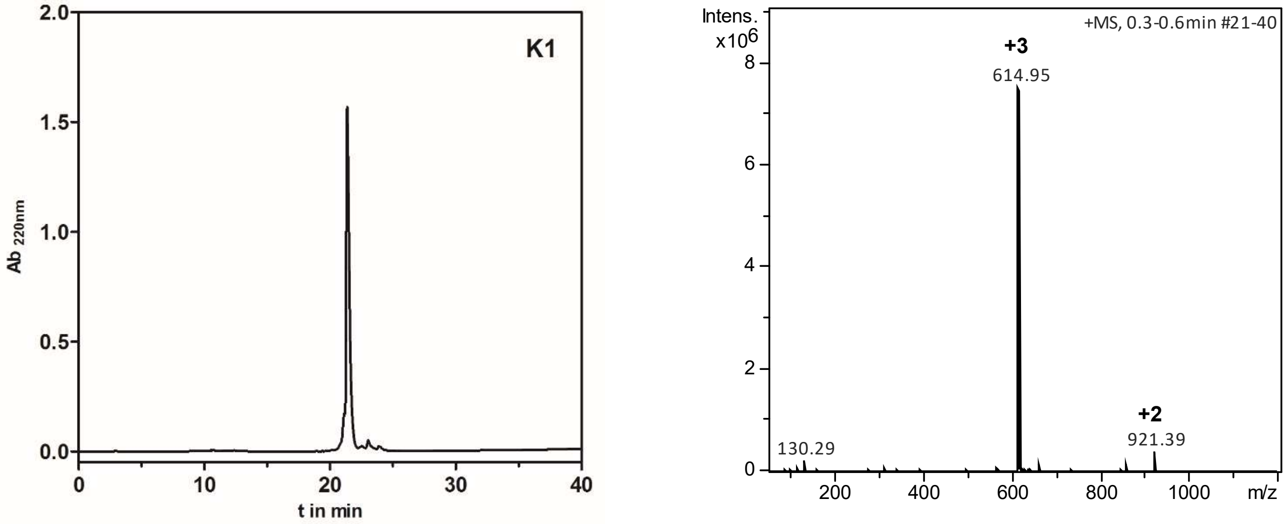

Figure S2. RP-HPLC profile and ESI-ion trap mass spectrum of GnRH-III-[4Ser, 8Lys(Dau=Aoa)] (**K1**). (MW_{cal}/MW_{exp} = 1841.89/1841.85 g/mol, *fragment ion: amino sugar loss of Dau)

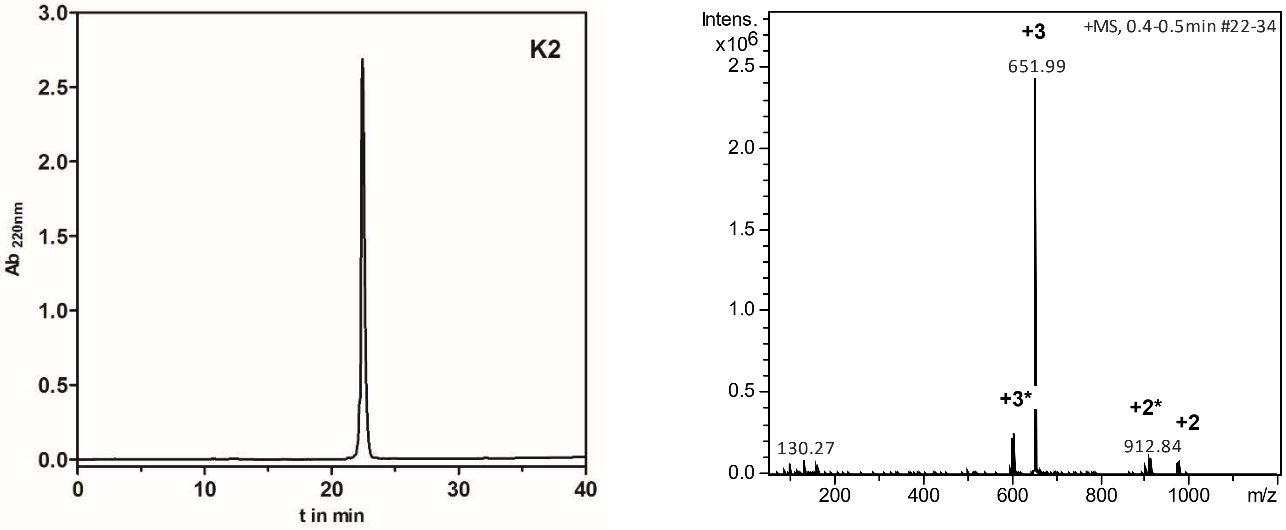

Figure S3. RP-HPLC profile and ESI-ion trap mass spectrum of GnRH-III-[4Lys(Bu), 8Lys(Dau=Aoa)] (**K2**). (MW_{cal} /MW_{exp} = 1953.07/1952.97 g/mol).

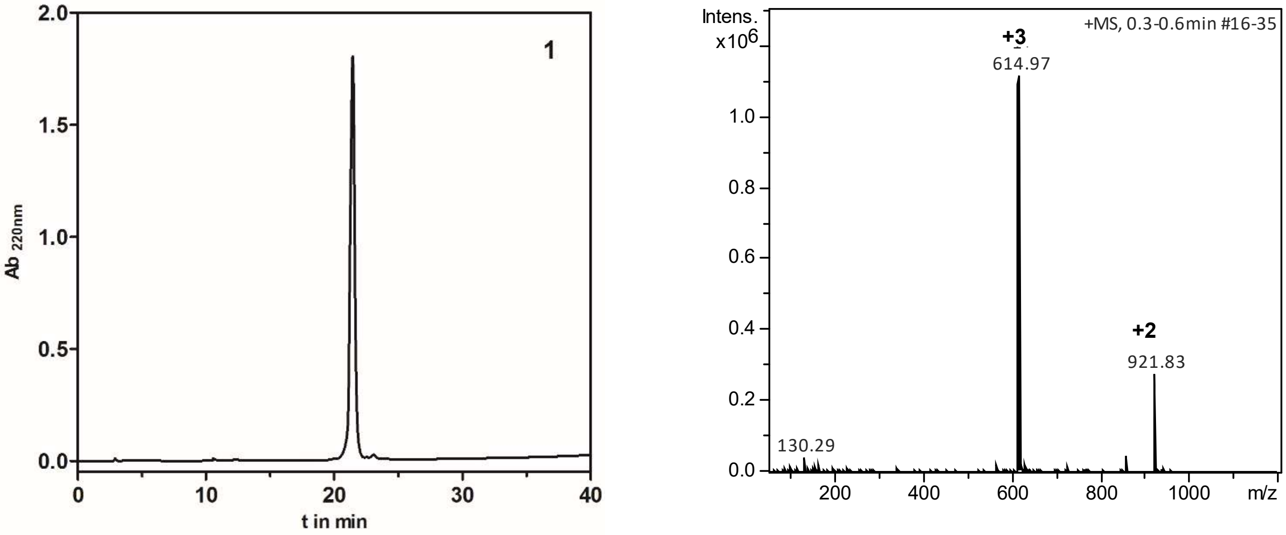

Figure S4. RP-HPLC profile and ESI-ion trap mass spectrum of GnRH-III-[3Trp, 8Lys(Dau=Aoa)] (**1**). (MW_{cal} /MW_{exp} = 1841.89/1841.91 g/mol).

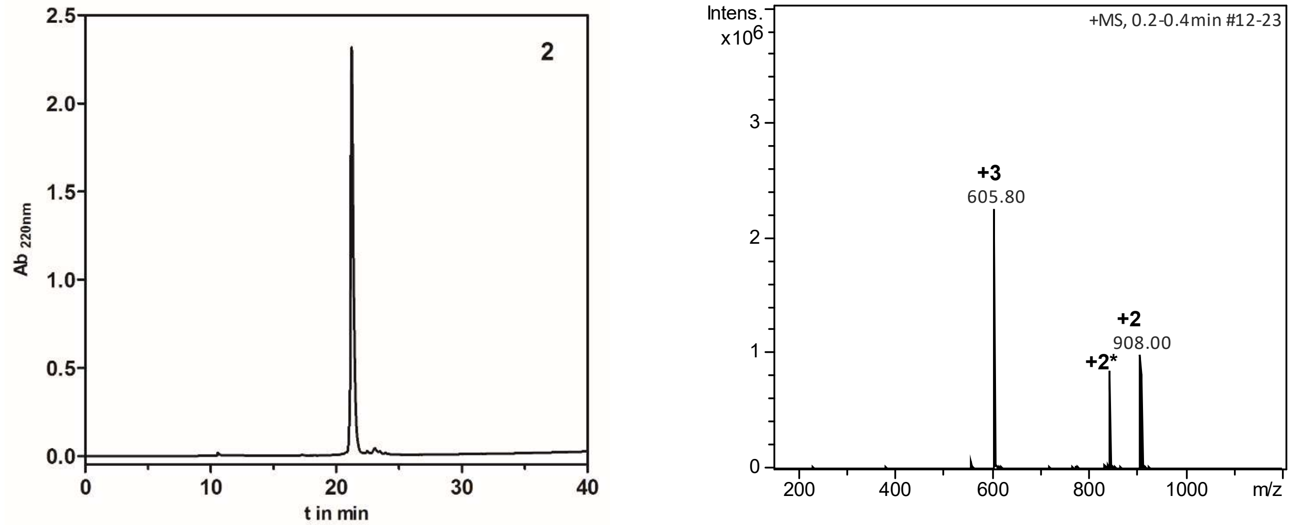

Figure S5. RP-HPLC profile and ESI-ion trap mass spectrum of GnRH-III-[³Tic, ⁸Lys(Dau=Aoa)] (**2**). (MW_{cal} /MW_{exp} = 1814.86/1814.40 g/mol).

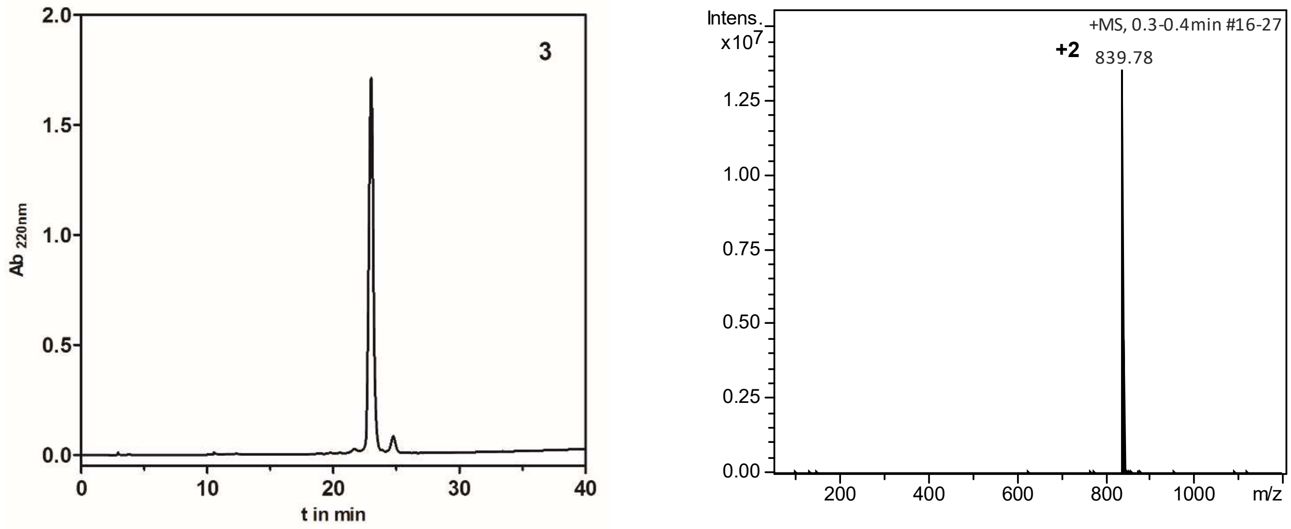

Figure S6. RP-HPLC profile and ESI-ion trap mass spectrum of GnRH-III-[²ΔHis, ³D-Tic, ⁸Lys(Dau=Aoa)] (**3**). (MW_{cal} /MW_{exp} = 1677.72/1677.56 g/mol).

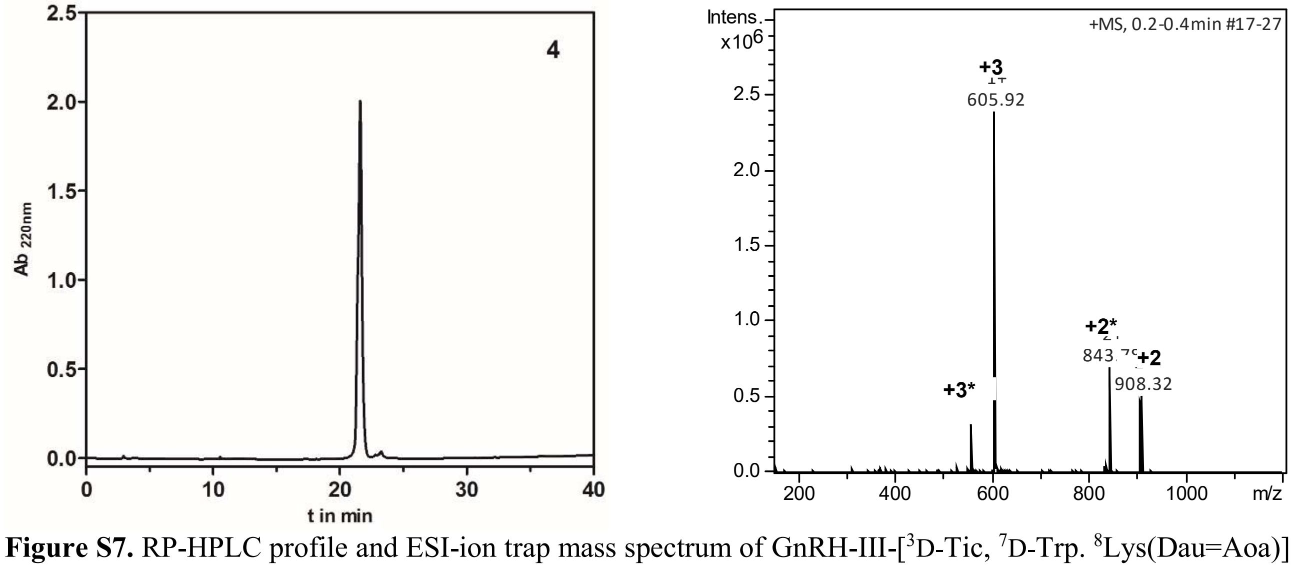

Figure S7. RP-HPLC profile and ESI-ion trap mass spectrum of GnRH-III-[³D-Tic, ⁷D-Trp. ⁸Lys(Dau=Aoa)] (**4**). (MW_{cal} /MW_{exp} = 1814.86/1814.64 g/mol).

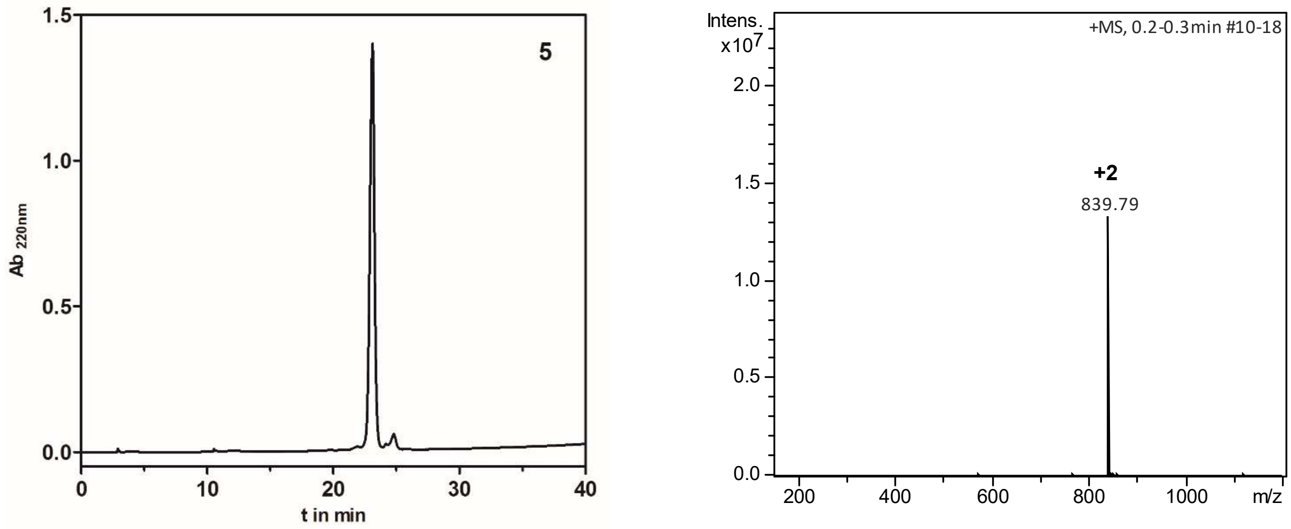

Figure S8. RP-HPLC profile and ESI-ion trap mass spectrum of GnRH-III-[$^{2}\Delta$His, ^{3}D-Tic, ^{7}D-Trp, ^{8}Lys(Dau=Aoa)] (**5**). (MW_{cal} /MW_{exp} = 1677.72/1677.58 g/mol).

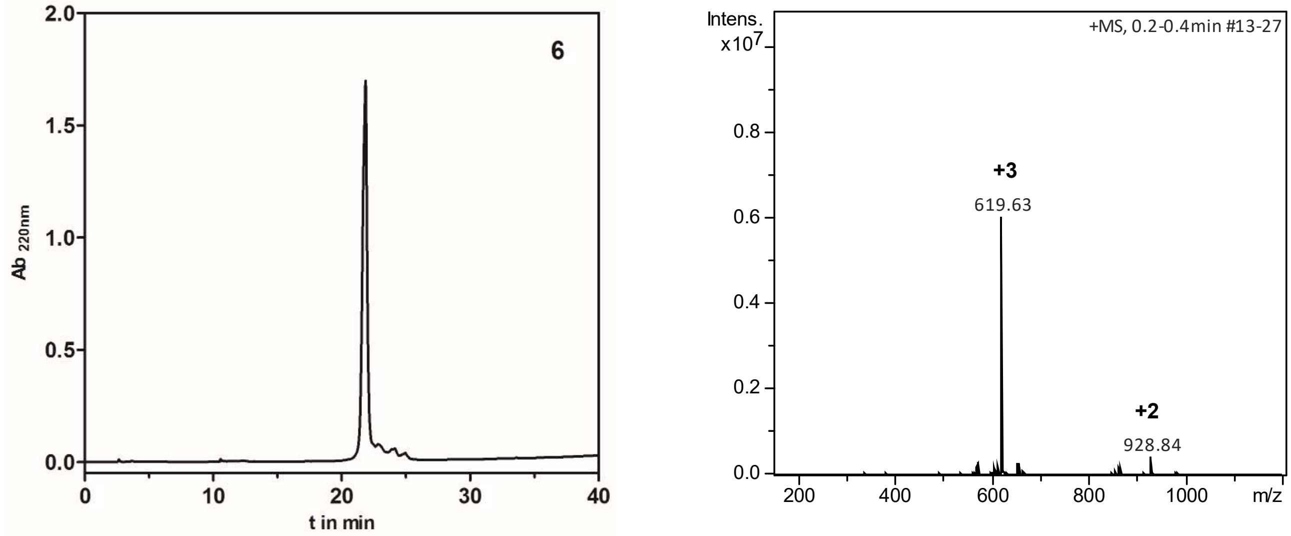

Figure S9. RP-HPLC profile and ESI-ion trap mass spectrum of GnRH-III-[^{6}Asp(OMe), ^{8}Lys(Dau=Aoa)] (**6**). (MW_{cal} /MW_{exp} = 1855.91/1855.68 g/mol).

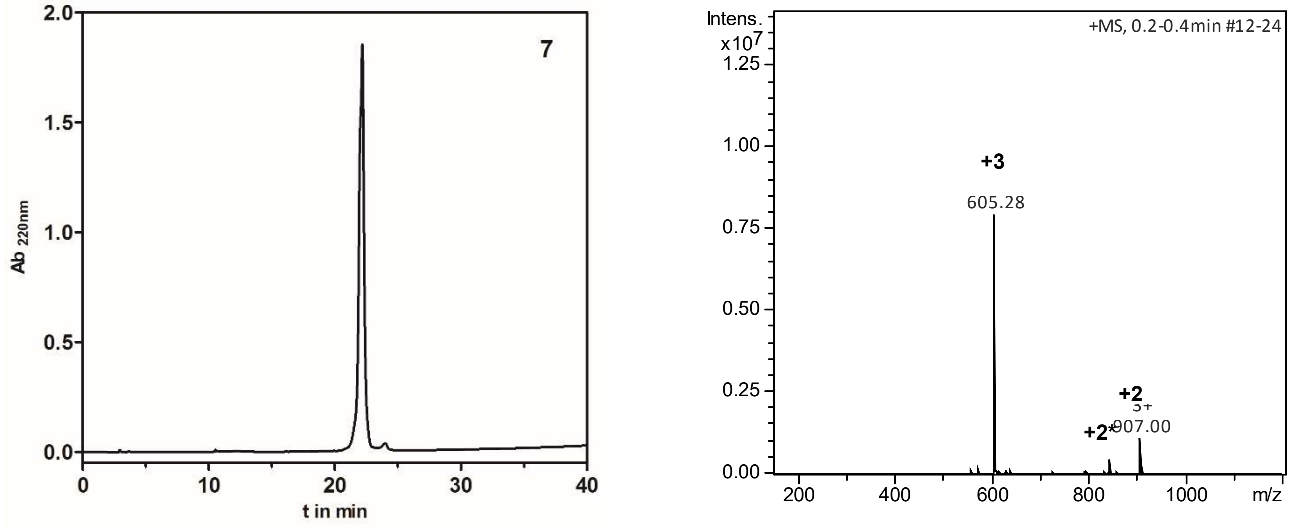

Figure S10. RP-HPLC profile and ESI-ion trap mass spectrum of GnRH-III-[^{8}Lys(Dau=Aoa), $^{10}\Delta$Gly-NH-Et] (**7**). (MW_{cal} /MW_{exp} = 1812.88/1812.84 g/mol).

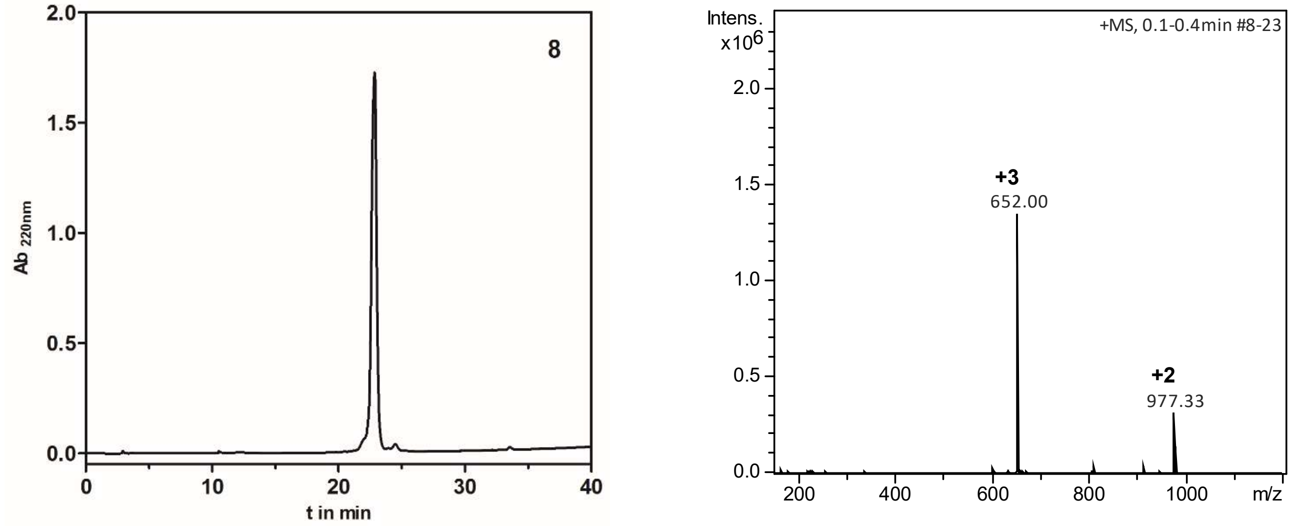

Figure S11. RP-HPLC profile and ESI-ion trap mass spectrum of GnRH-III-[[3]D-Trp, [4]Lys(Bu), [8]Lys(Dau=Aoa)] (**8**). (MW_{cal} /MW_{exp} = 1953.07/1953.00 g/mol).

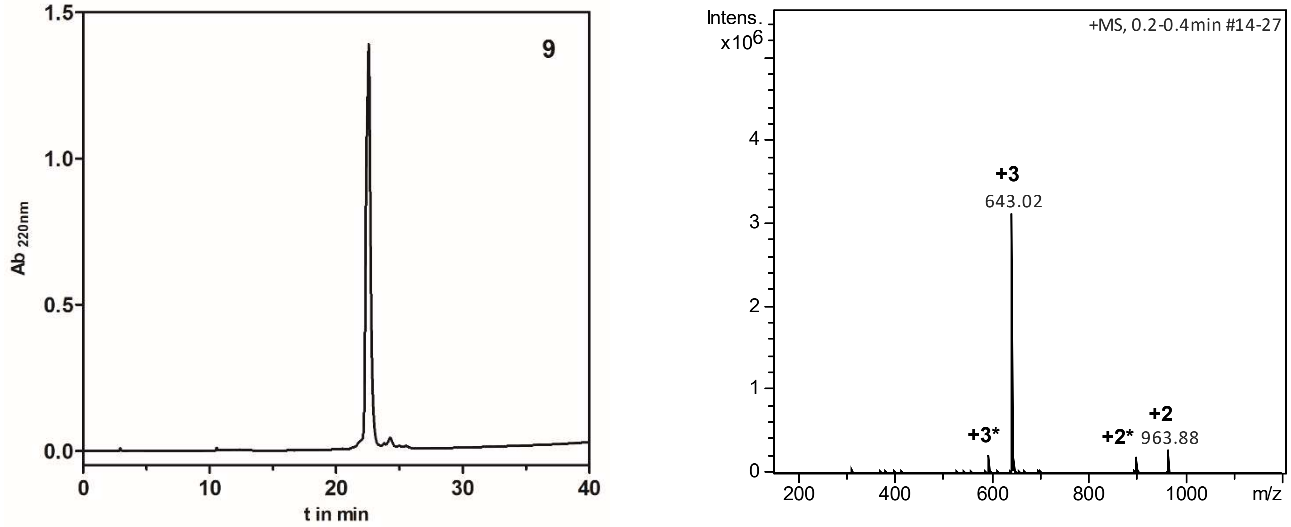

Figure S12. RP-HPLC profile and ESI-ion trap mass spectrum of GnRH-III-[[3]D-Tic, [4]Lys(Bu), [8]Lys(Dau=Aoa)] (**9**). (MW_{cal} /MW_{exp} = 1926.05/1926.06 g/mol).

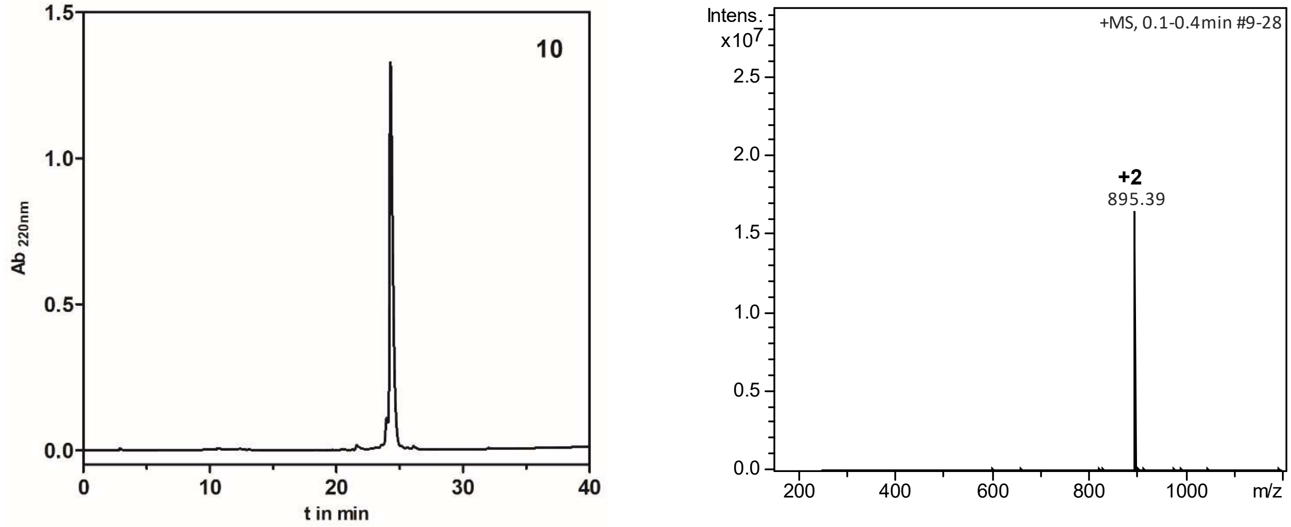

Figure S13. RP-HPLC profile and ESI-ion trap mass spectrum of GnRH-III-[[2]ΔHis, [3]D-Tic, [4]Lys(Bu), [8]Lys(Dau=Aoa)] (**10**). (MW_{cal} /MW_{exp} = 1788.91/1788.78 g/mol).

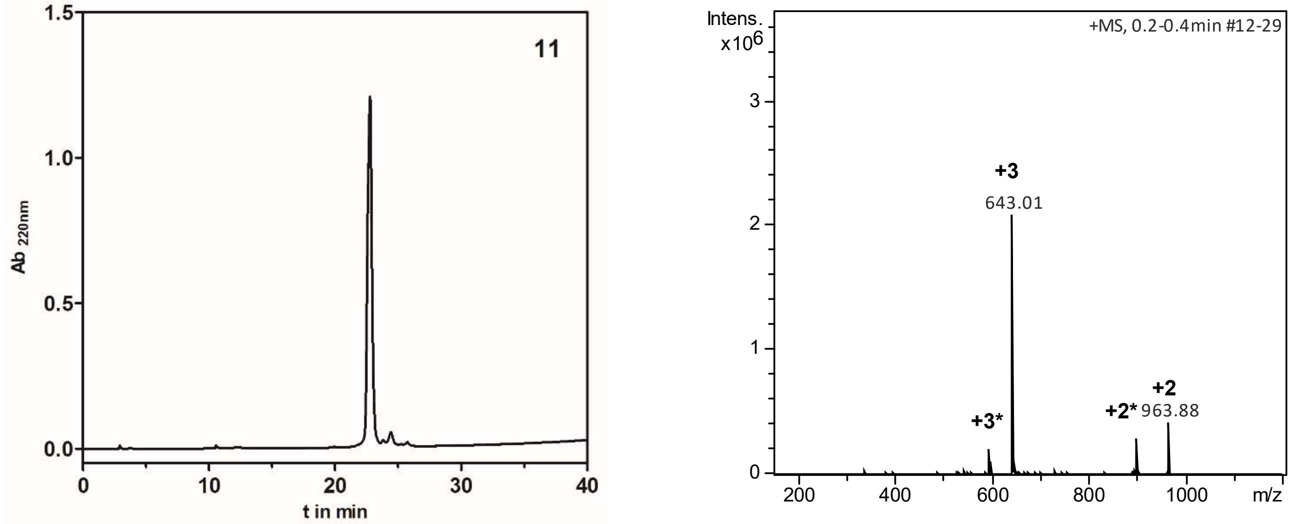

Figure S14. RP-HPLC profile and ESI-ion trap mass spectrum of GnRH-III-[, [3]D-Tic, [4]Lys(Bu), [7]D-Trp, [8]Lys(Dau=Aoa)] (**11**). (MW_{cal} /MW_{exp} = 1926.05/1926.03 g/mol).

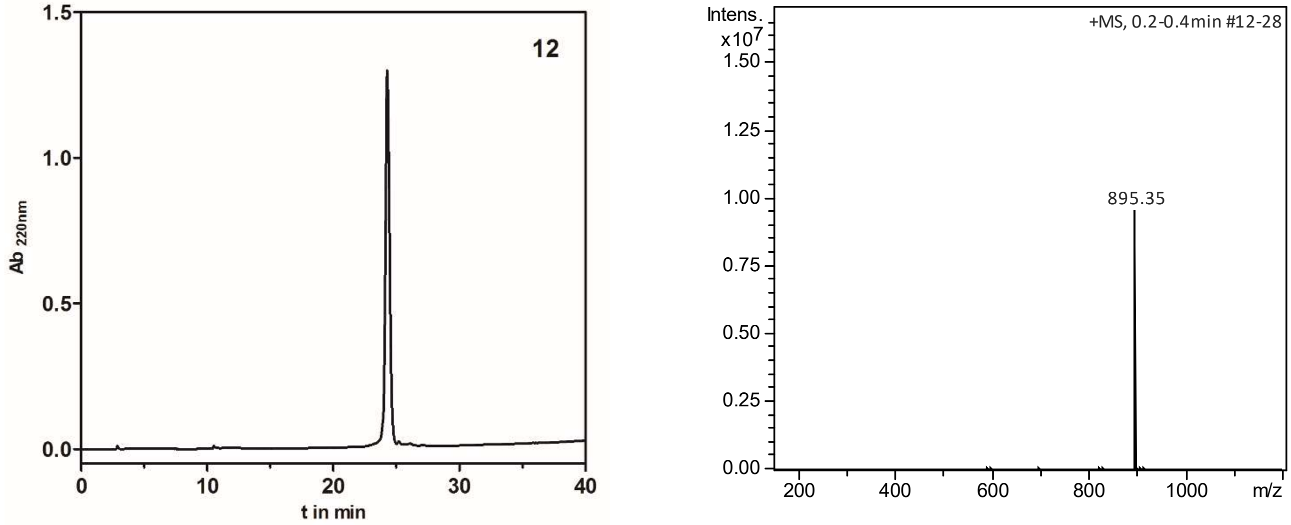

Figure S15. RP-HPLC profile and ESI-ion trap mass spectrum of GnRH-III-[[2]ΔHis, [3]D-Tic, [4]Lys(Bu), [7]D-Trp, [8]Lys(Dau=Aoa)] (**12**). (MW_{cal} /MW_{exp} = 1788.91/1788.70 g/mol).

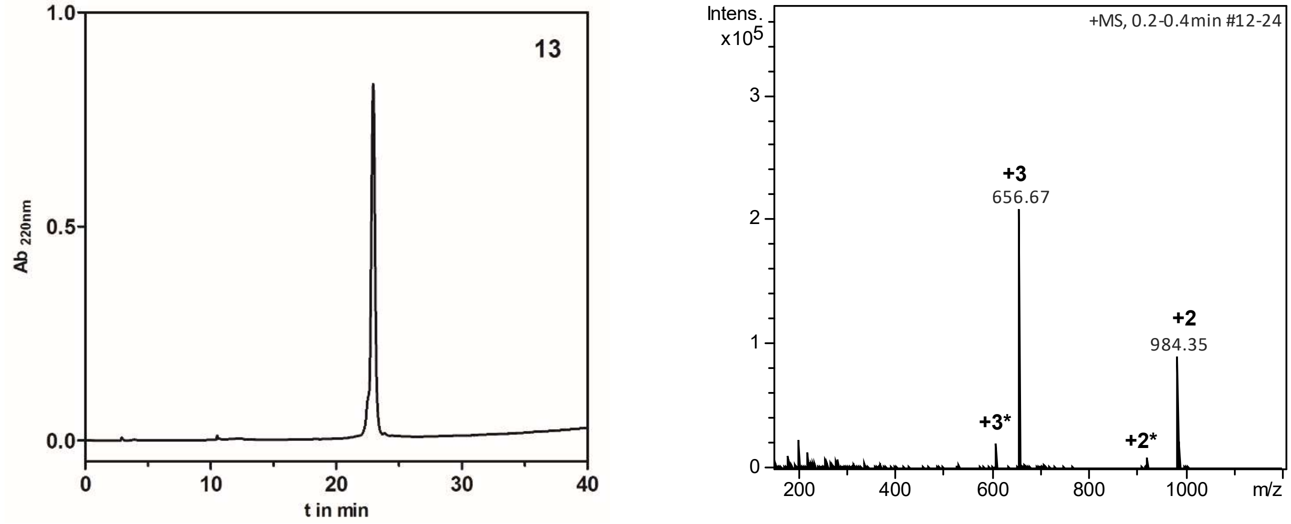

Figure S16. RP-HPLC profile and ESI-ion trap mass spectrum of GnRH-III-[[4]Lys(Bu), [6]Asp(OMe), [8]Lys(Dau=Aoa)] (**13**). (MW_{cal} /MW_{exp} = 1967.10/1967.01 g/mol).

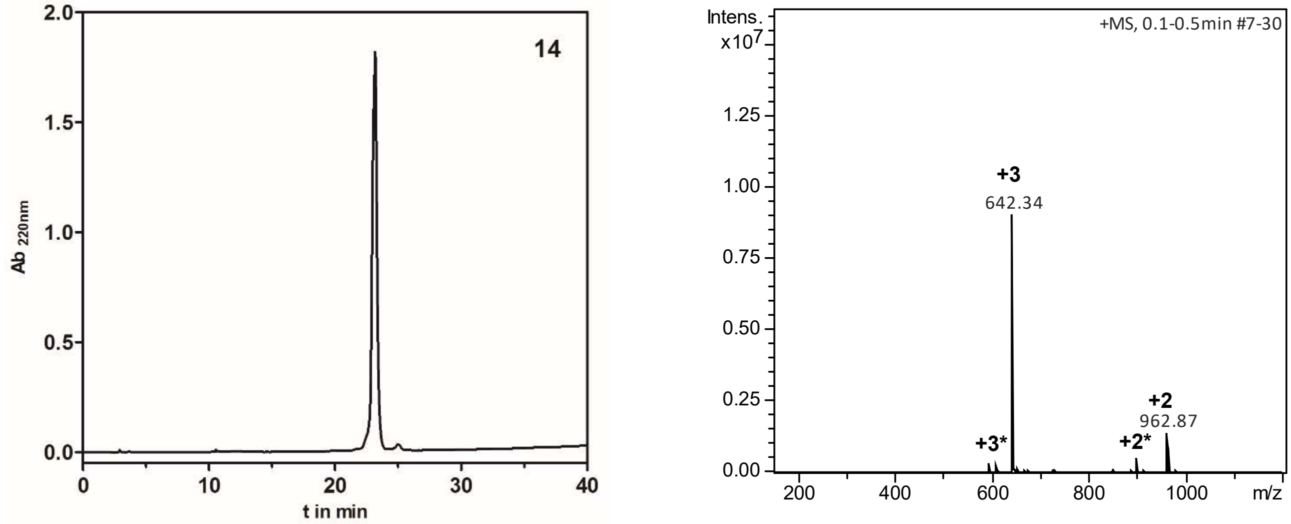

Figure S17. RP-HPLC profile and ESI-ion trap mass spectrum of GnRH-III-[[8]Lys(Dau=Aoa), [10]ΔGly-NH-Et] (**14**). (MW_{cal} /MW_{exp} = 1924.07/1924.02 g/mol).

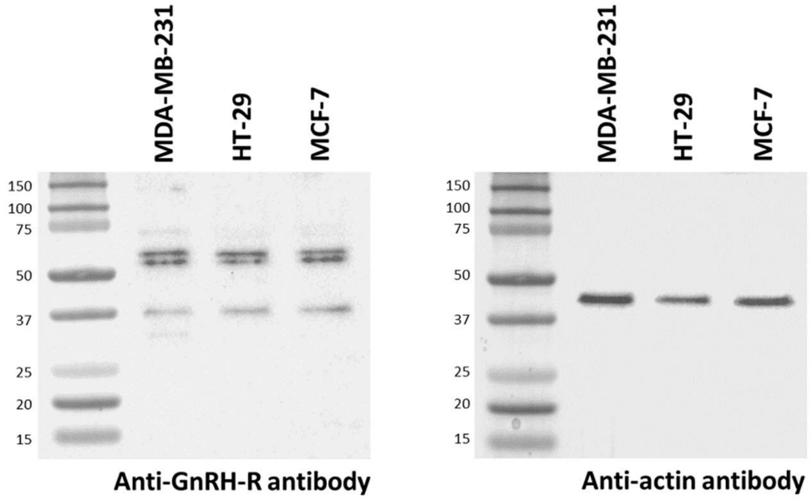

Figure S18. Western blot performed on whole cell lysates of MCF-7 and HT-29 cancer cells. Anti-GnRH-R antibody (Proteintech, 19950-1AP) (left). Actin expression was evaluated as loading control (Santa Cruz Biotechnology, sc-1616 (right). Band at 38 kDa represents the full length human GnRH-R; the signals at higher molecular weight (55-70 kDa) are assumed to be glycosylated forms of the GnRH-R.

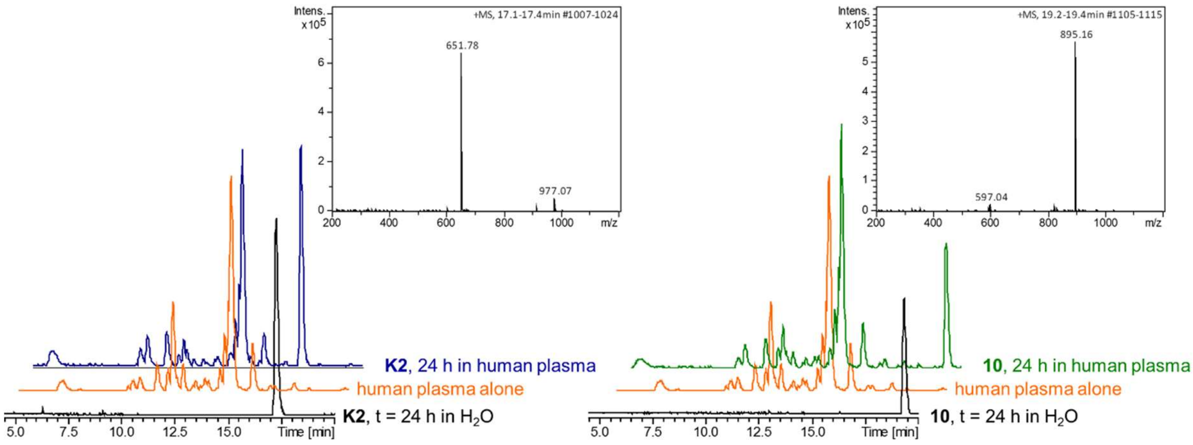

Figure S19. Stability of the bioconjugates **K2** and **10** in human plasma. LC-MS chromatogram of human plasma and the conjugates after 24 h incubation at 37 °C in H_2O or in human plasma plus the corresponding MS compound spectra.

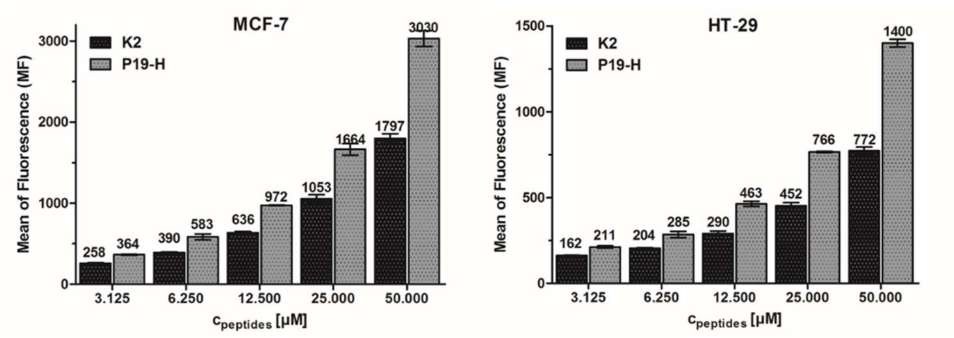

Figure S20. Mean of fluorescence of the cellular uptake of the GnRH-III conjugates **K2** and **10** on MCF-7 and HT-29 cancer cells after 6 h treatment determined by flow cytometry. The cellular uptake of **10** was significantly higher than the uptake of conjugate **K2** for both cells (paired Wilcoxon test, P=0.002516 and P=0.005099, respectively).

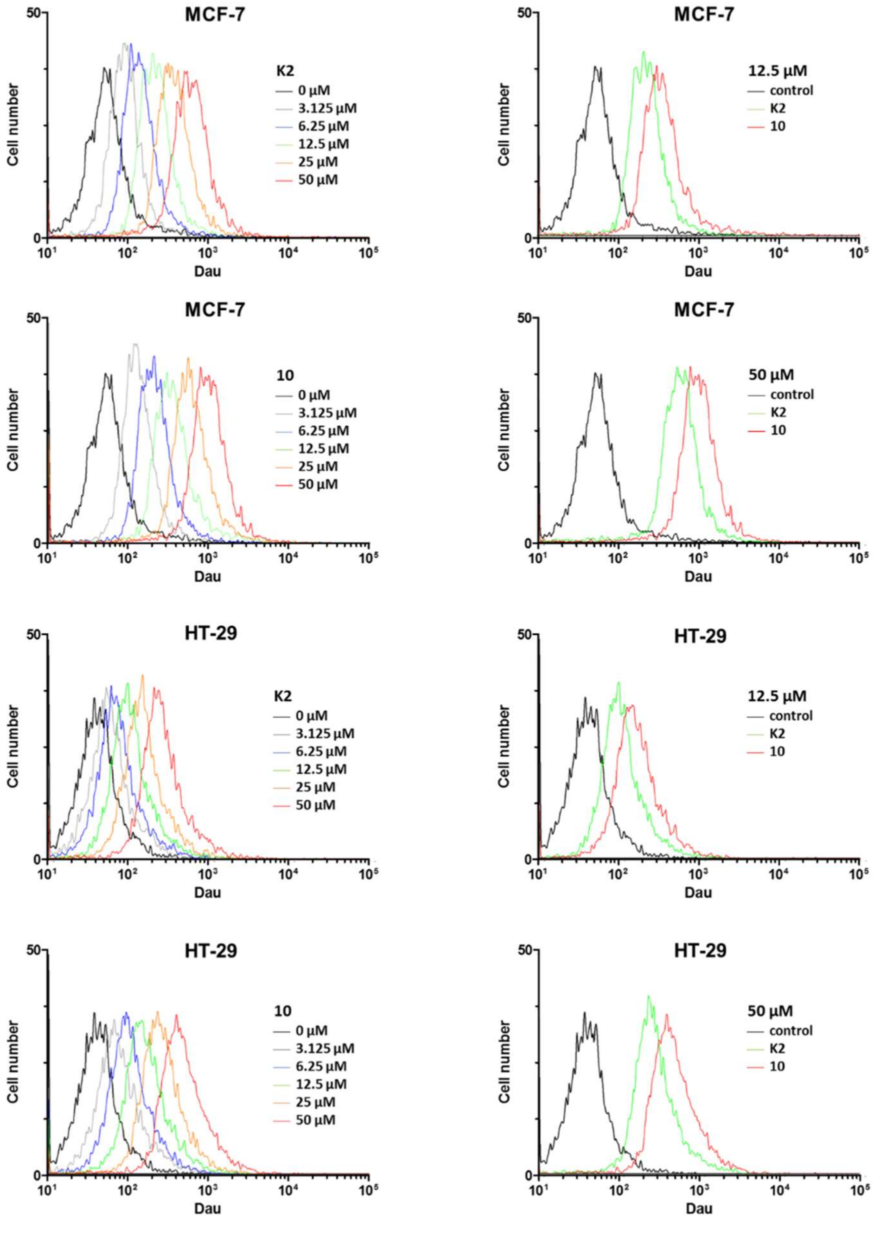

Figure S21. Cellular uptake of the GnRH-III conjugates **K2** and **10** on MCF-7 and HT-29 cancer cells after 6 h treatment determined by flow cytometry. FACS histograms show the concentration dependent cellular uptake.

Table S1. Fragments of GnRH-III-Dau conjugates **K2** and **10** produced by rat liver homogenate

Code	Compound	Fragment	MW$_{cal}$/MW$_{exp}$
**K2**	[^{4}Lys(Bu), ^{8}Lys(Dau=Aoa)]	<EHWK(Bu)HDWK(Dau=Aoa)PG-NH$_2$	1953.07/1952.79
		<EHWK(Bu)HDWK(Dau=Aoa)-OH	1799.92/1799.69
		H-HDWK(Dau=Aoa)PG-NH$_2$	1320.36/1319.95
		H-HDWK(Dau=Aoa)-OH	1167.18/1166.91
		H-K(Dau=Aoa)PG-NH$_2$	881.94/881.44
		H-K(Dau=Aoa)P-OH	825.86/825.40
		H-K(Dau=Aoa)-OH	728.75/728.37
		<EHWK(Bu)HD-OH	902.96/902.84
		<EHWK(Bu)-OH	650.73/650.71
		<EHW-OH	452.46/452.31
		H-DW-OH	319.32/319.27
**10**	[^{2}ΔHis, ^{3}D-Tic, ^{4}Lys(Bu), ^{8}Lys(Dau=Aoa)]	<E-D-Tic-K(Bu)HDWK(Dau=Aoa)PG-NH$_2$	1788.91/1788.33
		<E-D-Tic-K(Bu)HDWK(Dau=Aoa)-OH	1635.72/1635.22
		<E-D-Tic-K(Bu)HD-OH	738.78/738.26
		<E-D-Tic-K(Bu)H-OH	623.70/623.29
		<E-D-Tic-K(Bu)-OH	486,56/486.90
		H-K(Dau=Aoa)P-OH	825.86/825.40
		H-K(Dau=Aoa)-OH	728.75/728.37
